# Bufalin induces mitochondrial dysfunction and promotes apoptosis of glioma cells by regulating Annexin A2 and DRP1 protein expression

**DOI:** 10.1186/s12935-021-02137-x

**Published:** 2021-08-10

**Authors:** Yao Li, Yan Zhang, Xufang Wang, Qian Yang, Xuanxuan Zhou, Junsheng Wu, Xu Yang, Yani Zhao, Rui Lin, Yanhua Xie, Jiani Yuan, Xiaohui Zheng, Siwang Wang

**Affiliations:** 1 Faculty of Life Science & Medicine, Key Laboratory Resource Biology & Biotechnology in Western China, Ministry of Education, Northwest University, Xi’an, 710069 Shaanxi China; 2 Department of Acupuncture, Xi’an Hospital of Traditional Chinese Medicine, Xi’an, 710021 Shaanxi China; 3 Department of Chinese Materia Medica and Natural Medicines, Air Force Medical University, Xi’an, 710032 Shaanxi China; 4 Department of Pharmacy, Xijing Hospital, Fourth Military Medical University, Xi’an, 710032 Shaanxi China; 5 Air Force Hospital of Western Theater Command, Chengdu, 610083 Sichuan China

**Keywords:** Bufalin, Glioma cell, Mitochondrial dysfunction, DARTS-PAGE technology, Annexin A2, DRP1

## Abstract

**Background:**

Glioma is a common primary central nervous system tumour, and therapeutic drugs that can effectively improve the survival rate of patients in the clinic are lacking. Bufalin is effective in treating various tumours, but the mechanism by which it promotes the apoptosis of glioma cells is unclear. The aim of this study was to investigate the drug targets of bufalin in glioma cells and to clarify the apoptotic mechanism.

**Methods:**

Cell viability and proliferation were evaluated by CCK-8 and colony formation assays. Then, the cell cycle and apoptosis, intracellular ion homeostasis, oxidative stress levels and mitochondrial damage were assessed after bufalin treatment. DARTS-PAGE technology was employed and LC–MS/MS was performed to explore the drug targets of bufalin in U251 cells. Molecular docking and western blotting were performed to identify potential targets. siRNA targeting Annexin A2 and the DRP1 protein inhibitor Mdivi-1 were used to confirm the targets of bufalin.

**Results:**

Bufalin upregulated the expression of cytochrome C, cleaved caspase 3, p-Chk1 and p-p53 proteins to induce U251 cell apoptosis and cycle arrest in the S phase. Bufalin also induced oxidative stress in U251 cells, destroyed intracellular ion homeostasis, and caused mitochondrial damage. The expression of mitochondrial division-/fusion-related proteins in U251 cells was abnormal, the Annexin A2 and DRP1 proteins were translocated from the cytoplasm to mitochondria, and the MFN2 protein was released from mitochondria into the cytoplasm after bufalin treatment, disrupting the mitochondrial division/fusion balance in U251 cells.

**Conclusions:**

Our research indicated that bufalin can cause Annexin A2 and DRP1 oligomerization on the surface of mitochondria and disrupt the mitochondrial division/fusion balance to induce U251 cell apoptosis.

**Graphic Abstract:**

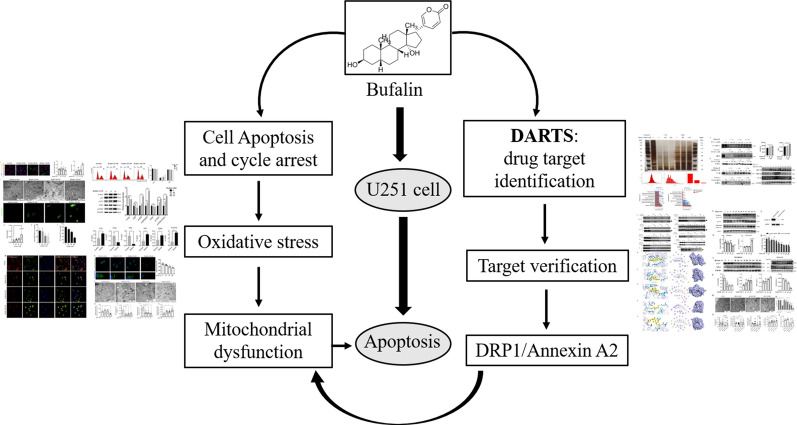

**Supplementary Information:**

The online version contains supplementary material available at 10.1186/s12935-021-02137-x.

## Introduction

Glioma, an intracranial primary tumour, is the most common malignancy in the central nervous system, accounting for approximately 50% to 60% of intracranial primary tumours, and highly malignant glioblastoma accounts for approximately 45% of all types of glioma [1, 2]. Because of its invasive growth, glioma can invade normal brain tissue surrounding the tumour. After traditional surgical resection, the recurrence rate is very high. Therefore, in addition to surgery, a combination of radiotherapy and chemotherapy is administered as a standard clinical treatment method for damaging and destroying the DNA of glioma cells. Temozolomide is an alkylating antitumour drug with high oral bioavailability that easily penetrates the blood–brain barrier [3]. It is currently the first-line drug for the clinical treatment of glioma. After temozolomide enters tumour cells, its decomposition products can cause DNA methylation, which interferes with cell DNA replication, causes DNA damage, and achieves antitumour effects [4]. However, the use of temozolomide in the treatment of gliomas is likely to lead to drug resistance, and therefore, the clinical effective rate of temozolomide administration for glioma patients is less than 45% [5]. Hence, it is important to find a new drug that can target the treatment of glioma effectively.

Drug affinity responsive target stability (DARTS) technology was first proposed and studied by Lomenick in 2009 [6]. Researchers have since found that specific DNA-binding sites bound by their corresponding transcription factors endow cells with anti-DNase degradation. Therefore, it has been hypothesized that a drug targeted protein may be resistant to protease hydrolysis after binding to the drug. For example, the complex obtained by coincubation of FKBP12-rapamycin and FK506 was not destroyed by subtilisin, which confirms the feasibility of the DARTS method. Hence, DARTS has been recently used in drug target identification research [7].

Bufalin (Additional file 1: Fig. S1) is the main active ingredient of Chan Su, which is extracted from the venom of *Bufo gargarizans* Cantor or *Bufo melanostictus* Schneider. Its chemical name is 3β,14-dihydroxy-5β,20(22)-bufadienolide, and the content in dry toad venom can be as high as 1%–5% [8]. Numerous studies have shown that bufalin has an excellent inhibitory effect on prostate cancer cells, cervical cancer cells, leukaemia cells, non-small cell lung cancer cells and glioma cells [9–12]. Bufalin has been reported to have digoxin-like effects, which indicates that it inhibits the activity of Na^+^/K^+^-ATPase [13]. Researchers have found that bufalin can bind to Na^+^/K^+^-ATPase, inhibit the sodium potassium pump, and further increase the intracellular calcium ion concentration, causing internal plasma reticulum stress and eventually triggering cell apoptosis [14]. Bufalin can inhibit the proliferation, colony formation and stem cell-like phenotype of U87 and U251 cells by increasing the expression of miR-203 [15]. Bufalin can also upregulate the expression of apoptotic proteins such as cleaved caspase 3 and poly(ADP ribose) polymerase, downregulate the expression of telomerase reverse transcriptase, induce apoptosis of glioma-like stem cells, and increase the sensitivity of glioma cells to temozolomide [16]. However, the mechanism by which bufalin induces oxidative stress-mediated mitochondrial dysfunction leading to cell apoptosis is unclear. Therefore, we used DARTS-PAGE technology combined with silver staining, LC–MS/MS and molecular biology techniques to further study the molecular mechanism of bufalin antiglioma effects and to provide experimental evidence for its clinical application.

## Materials and methods

### Cell culture and reagents

The U251 cell line was purchased from Shanghai Cell Bank (Shanghai, China), cultured in high-glucose DMEM (Thermo Fisher, USA) supplemented with 10% foetal bovine serum (FBS) and 1% antibiotics (100 U/mL penicillin G and 0.1 mg/mL streptomycin), and then maintained in the exponential growth phase in an atmosphere of 5% CO_2_ at 37 °C. Bufalin (≥ 98% in purity) was purchased from Baoji Chenguang Technology Development Co., Ltd. (Baoji, Shaanxi, China).

### Cell viability assay

Cell counting kit-8 (Dojindo Laboratories, Tokyo, Japan) was used to detect cell viability. U251 cells (5 × 10^3^ cells/well) were seeded in 96-well plates and cultured overnight. The culture medium was replaced with medium containing different concentrations of bufalin, and 10% FBS culture medium (0.1% DMSO) was added to the control well. After incubation for 12 h, 24 h, and 48 h, the culture medium was discarded, and 100 µL of CCK-8 working solution was added.

### Clone formation assay

U251 cells treated with 25 nM, 50 nM, and 100 nM bufalin for 24 h were seeded on new 6-well plates at a density of 100 cells/well and then incubated at 37 °C in 5% CO_2_ for 1 week. After staining with Giemsa (Solarbio Science & Technology, Beijing, China), the clone formation rate was calculated as follows: clone formation rate (%) = numbers of clones/numbers of inoculated cells × 100%.

### Cellular ATP content detection

The ATP assay (Merck, Darmstadt, Germany) was used to detect cellular ATP content. Cells treated with different concentrations of bufalin were digested with trypsin, collected, and centrifuged for 5 min at 1500 rpm. A 10 µL cell suspension consisting of 10^4^ cells was transferred onto a luminometer plate, and 100 µL of nucleotide lysate was added. After 5 min, 10 µL of ATP detection working solution was added to the cell lysate, and the luminescence level of each well was measured within 1 to 2 min with a fluorescence microplate reader. The steps in the reader manufacturer’s instructions were followed for drawing a standard curve and calculating the ATP content of each well.

### Na^+^/K^+^ -ATPase activity assay

Cells treated with 25 nM, 50 nM, and 100 nM bufalin were collected after 24 h in culture, and 1 mL was extracted and added to each tube (adjusting cells at a density of 1 × 10^6^ cells/mL in each group). The cells were sonicated at 20% power, with ultrasound applied for 3 s with 10 s intervals, and this cycle was repeated 30 times. Then, the cells were centrifuged for 10 min at 8000 g at 4 °C. The supernatant was transferred to 2-mL centrifuge tubes, and the enzymatic reaction and phosphorus levels were measured according to the instructions of an Na + /K + -ATPase activity kit (Solarbio Science & Technology, Beijing, China). Samples (200 µL) were placed in 96-well plates, and the absorbance was measured at 660 nm.

### Intracellular Ca^2+^ level assay

Intracellular calcium ion levels were measured according to the Fluo-3AM manufacturer’s instructions. The Fluo 3-AM storage solution (1 mM) was prepared by dissolving 50 mg Fluo 3-AM powder (Dojindo Laboratories, Japan) with 44.2 μL of DMSO and stored at -20 °C in the dark. Furthermore, a Fluo 3-AM working solution (5 μM) was prepared by dissolving 44.2 μL of Fluo 3-AM storage solution and 16.2 μL Pluronic® F-127 (20% solution in DMSO, Invitrogen®, Thermo Fisher Scientific) with 8.80 mL of Hanks' Balanced Salt Solution (Gibco® HBSS, Thermo Fisher Scientific). Briefly, the cells were seeded in confocal dishes, incubated overnight and then exposed to bufalin in fresh culture medium at concentrations of 25 nM, 50 nM, and 100 nM. After 24 h of bufalin treatment, the cells were washed three times with PBS and inoculated with Fluo 3-AM working solution at 1.5 mL/dish for 45 min in a dark cell incubator and then washed with PBS. The cells were continuously incubated with HBSS at 37 °C with 5% CO_2_ for 20 min to ensure the complete de-esterification of Fluo 3-AM. The fluorescence intensities were measured at λ_ex_/λ_em_ = 490/520 nm by an Olympus FV1000 confocal microscope (Olympus; Center Valley, PA, USA).

### Reactive oxygen species (ROS) assay

The reactive oxygen species (ROS) detection storage solution was prepared by dissolving ROS detection reagent (Sigma-Aldrich, St. Louis, MO, USA) with 40 μL of DMSO and stored at 4 °C in the dark. Five thousand cells were inoculated in a confocal culture dish. After they adhered to the dish walls, the cells were exposed to 25, 50 and 100 nM bufalin for 24 h. Two millilitres of ROS detection reagent in working solution was added to each dish and incubated at 37 °C for 40 min in the dark. The cells were washed 3 times with PBS solution, and 2 mL of PBS solution was added to each dish to cover the cells, and the fluorescence intensities were measured at λ_ex_/λ_em_ = 490/520 nm with an Olympus FV1000 confocal microscope (Olympus; Center Valley, PA, USA).

### Glutathione (GSH) assay

The intracellular GSH level was measured by a GSH/GSSG-Glo assay kit (Solarbio Science & Technology, Beijing, China). U251 cells treated with different concentrations of bufalin for 24 h were collected, and the contents of GSH and GSSG in each group of cells were determined according to the instructions of the GSH and GSSG activity assay kit. Then, the GSH/GSSG ratio was calculated.

### Flow cytometry (FCM)

Cells were collected in aseptic tubes after drug treatment, 490 μL of assay buffer, 5.0 μL of Annexin V labelled with fluorescein (FITC-Annexin V) and 5.0 μL of propidium iodide (PI) were added, and the cells were incubated for 20 min and detected by FCM. According to the staining results, the proportion of living cells (Annexin V-/PI-), early apoptotic cells (Annexin V + /PI-), late apoptotic cells and necrotic cells (Annexin V + /PI +) were distinguished in each group.

Cell cycle analysis was carried out by FCM. Through the combination of PI and DNA, FCM was used to categorize cell stage directly on the basis of the fluorescence intensity indicating DNA content. G1/G0 phase cells have DNA content of diploid cells, G2/M phase cells have DNA content of tetraploid cells, and S phase cells have DNA content of both diploid and tetraploid cells. After bufalin treatment, the cells were collected, fixed with 70% ethanol and incubated overnight at 4 °C. After PBS washes, PI staining solution containing RNA enzyme was added to the tubes containing the cells. The cells were incubated at 37 °C for 30 min and subsequently subjected to FCM.

### Cell apoptosis analysis

A MitoTracker red CMX Ros kit (Beyotime Biotechnology, Shanghai, China) was used to identify apoptotic U251 cells treated with 25, 50 or 100 nM bufalin for 24 h. An Olympus FV1000 confocal microscope was used to detect red fluorescence at λ_ex_/λ_em_ = 579/599 nm, green fluorescence at λ_ex_/λ_em_ λex/λem = 492/520 nm, and blue fluorescence at λ_ex_/λ_em_ = 350/461 nm.

### Transmission electron microscopy (TEM)

The cells treated with different concentrations of bufalin for 24 h were digested and collected in a centrifuge tube. After the cells were washed twice with PBS, 2.5% glutaraldehyde solution was added along the tube wall to cover the cell clumps, and the cells were incubated in these tubes overnight at 4 °C. The cells were then washed 2 times with PBS, fixed with 1% osmium acid, dehydrated in a gradient, and embedded in resin that was then cut into ultrathin sections and stained with 2% uranyl acetate for 5 min. The ultrastructural changes of the cells were observed by TEM.

### Mitochondrial membrane potential (MMP) assay

JC-10 is a cationic lipophilic dye that forms reversibly emitting red fluorescence when concentrated in cells with polarized mitochondrial membranes. When the mitochondrial membrane potential decreases and JC-10 cannot accumulate in the matrix of mitochondria, JC-10 is a monomer that emits green fluorescence. By calculating the ratio of red/green fluorescence, the MMP level in U251 cells was determined to evaluate the effect of bufalin on cell mitochondrial function. The MMP was determined using a mitochondria membrane potential kit (Sigma-Aldrich, St. Louis, MO, USA), which contains JC-10 dye. U251 cells were added to confocal Petri dishes (1 × 10^5^ cells/dish) and treated with 25, 50 or 100 nM bufalin for 24 h. Then, the medium was discarded, and the cells were washed with PBS, 2 mL of JC-10 working solution was added to each dish to cover the cells, and the cells were incubated at 37 °C in the dark for 40 min. Then, the cells were washed with PBS, the fluorescence intensity was observed under a confocal microscope, and the ratio of red/green fluorescence was calculated.

### RT-QPCR

Total RNA was extracted using TRIzol reagent. cDNA was synthesized using TaqMan™ reverse transcription reagents (Applied Biosystems, Foster City, CA). Quantitative reverse transcriptase-polymerase chain reaction (RT-QPCR) analyses were carried out to determine the mRNA levels (Chk1, Chk2, ATM, ATR, CDK2 and CDC25A mRNA) using SYBR Green Real-Time PCR Master Mixes (Applied Biosystems). β-Actin was the internal control. The primers used for RT-QPCR are shown in Additional file 1: Table S1; they were designed by Shanghai Novelbio Medical Technology Co., Ltd.

### Western blot analysis

Proteins from cells treated with bufalin for 24 h or protein samples taken after bufalin incubation with U251 cell were separated by SDS electrophoresis. The following primary antibodies were used for western blotting: anti-p53 (#9282), anti-p-p53 (#9284), anti-Chk1 (#2360), anti-p- anti-Chk1 (#2344), anti-c-myc (#13,987), anti-cytochrome C (#11,940), anti-caspase 3 (#9662) and anti-cleaved caspase 3 (#9661) (Cell Signaling Technology, Danvers, MA, USA); anti-DRP1 (WL03028), anti-TUBb (WL01931), anti-Annexin A2 (WL0033a) and anti-COX IV (WL02203) (Wanleibio Technology, Shenyang, China); anti-HSPA8 (E-AB-22118), anti-HSPA9 (E-AB-11284), anti-mitochondrial fusion protein-2 (E-AB-32025) (Elabscience, Wuhan, China) and anti-GAPDH (G9545) (Sigma-Aldrich, St. Louis, USA), which was used as the internal control. After treatment with primary antibodies, the membrane was treated with the appropriate secondary antibody conjugated to horseradish peroxidase (HRP; Santa Cruz). Western blotting was performed three times. The intensity of each band was quantified with ImageJ software.

### Drug affinity responsive target stability (DARTS) assay

Total protein in U251 cells was extracted, 66 μL of 10 × TNC solution was added to 600 μL of total protein after BCA quantification. The protein in TNC solution was divide into three aliquots, and 2.0 μL of 100 μM DMSO and 1000 μM bufalin were added to each aliquot, which was mixed gently and incubated overnight in a refrigerator at 4 °C. Fifty microlitres of the cell protein extract combined with bufalin was added to 2.0 μL of 1.25 mg/mL 1 × TNC solution and 0.25 mg/mL pronase working solution, enzymatically digested at room temperature for 15 min, and then added to 5 × loading buffer for denaturation. The proteins were separated by SDS-PAGE electrophoresis, and LC–MS/MS analysis was performed after silver staining in accordance with the protocol of a Pierce silver stain kit (Thermo Fisher, USA).

### LC–MS/MS and proteomics analysis

A SCIEX triple TOF 5600 LC–MS/MS system was used to perform mass spectrometry analysis of the different bands, and the peptide samples bound to the C18 capture column were gradient eluted for analysis. Ultrapure water with 0.1% formic acid (A) and acetonitrile with 0.1% formic acid (B) constituted the mobile phase, and the gradient elution programmes were as follows: 0 min-15 min, 5%–35% B; 15 min-16 min, 35%–80% B; 16 min-21 min, 80% B; 21 min-21.1 min, 80%–5% B; and 21.1 min-29 min, 5% B. The flow rate was 0.3 µL/min. Mass spectrometry IDA mode analysis included one full MS scan (at m/z 350–1500, 250 ms) in each scan cycle, followed by 40 MS/MS scans (at m/z 100–1500, 50 ms). MS/MS collected any precursor ion signal greater than 120 cps, the charge number was + 2– + 5, and the exclusion time of repeated ion collection was set to 18 s.

The mass spectrum data were retrieved by ProteinPilot (V4.5), the Paragon database retrieval algorithm was used, and the human proteome reference database in UniProt was referenced. The search results were screened with unused ≥ 1.3 as the standard, the entries and contaminating proteins retrieved with an anti-database were deleted, and the remaining identification information was assessed in a followed-up analysis.

### Molecular docking

The MOE-DOCK module was used to dock and predict the affinity of the ligand and the receptor. In this procedure small-molecule drugs are defined as ligands, and proteins are defined as receptors. The 3D structures of the Annexin A2, TUBb, DRP1, HSPA9 and HSPA8 proteins were downloaded from the RCSB Protein Data Bank (http://www.rcsb.org/). With LigX, the protonation state and hydrogen orientation of the protein were optimized at pH 7 and 300 K. The docking process adopts a flexible induced fit mode, the side chain in the amino acid binding pocket can be optimized and adjusted according to the ligand conformation, and the weight of restrained side chain rotation was set to 10. For each ligand, a total of 1000 conformations were obtained, and all docking poses were ranked by London dG scoring, and the top 30 poses were then rescored by the GBVI/WSA dG method. Finally, the representative conformation was selected based on the binding score. The interaction mode of ligand and receptor was mapped by PyMOL software (http://www.pymol.org).

### Small interfering RNA transfection

U251 cells were seeded in a 6-well plate at a density of 1 × 10^6^ cells/well. LipofectamineTM 2000 reagent was used to transfect Annexin A2 siRNA (GenePharma, Shanghai, China) with the sequence 5'-TGTGTGGTGGAGATGACTGA-3' into U251 cells to represent transfection into humans. A genomic sequence without a matching negative siRNA was used as the negative control, and U251 cells treated only with LipofectamineTM 2000 reagent were used for the mock control group. Seventy-two hours after Annexin A2 siRNA was transfected into U251 cells, western blotting was performed to detect the expression level of Annexin A2 protein in cells.

### Statistical analysis

Each experiment was performed at least three times and analysed by GraphPad Prism 7 software. The data are expressed as the means ± SD. *P* values were calculated using one-way ANOVA when the variances were uniform, and when the variances were not uniform, nonparametric tests were performed for statistical analysis.* P* < 0.05 was considered statistically significant.

## Results

### Bufalin induces U251 cell apoptosis and intracellular oxidative stress

To examine the effect of bufalin in U251 cells, we treated the U251 cell line with different concentrations of bufalin for 12 h, 24 h and 48 h and performed CCK-8, colony formation and cell apoptosis assays. The results showed that bufalin has a significant proliferation inhibitory effect on U251 cells, which can lead to loss of cell viability and colony formation and increase the proportion of the apoptotic cell population (Additional file 1: Fig. S2 A, B, and F). Fluo 3-AM staining and Na^+^/K^+^-ATPase activity kits were employed to detect the Na^+^/K^+^-ATPase activity and Ca^2+^ content in U251 cells after bufalin treatment. The results showed that bufalin can inhibit Na^+^/K^+^-ATPase activity and increase the Ca^2+^ levels in U251 cells in a dose-dependent manner (Additional file 1: Fig. S2 C, D, and E).

**Fig. 1:**
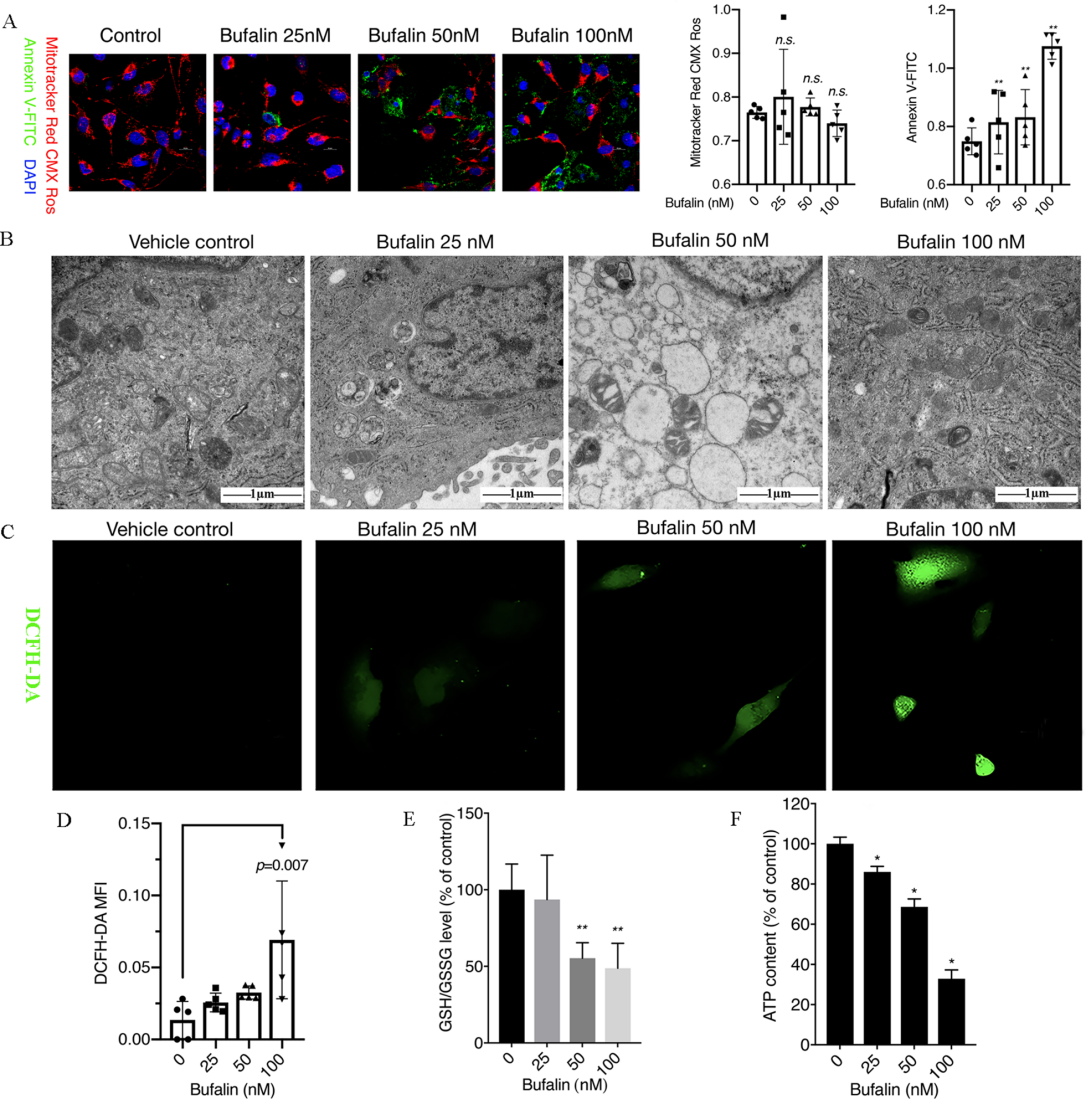
Bufalin induces cell apoptosis and oxidative stress. **A** U251 cells were treated with 25, 50 or 100 nM bufalin for 12 h, and cell apoptosis was observed by confocal microscopy after MitoTracker Red CMX ROS, Annexin V-FITC and Hoechst 33,342 co-staining (n = 3). The Y axis in MitoTracker Red CMX Ros and Annexin V-FITC plot represents the intensity of red and green fluorescence. **B** Ultrastructure of U251 cells as detected with TEM. **C** The intracellular ROS content was observed by laser confocal microscopy after DCFH-DA staining (n = 3). **D** The ROS levels in U251 cells. **E** The ratio of GSH/GSSH in each group of cells, **p < 0.01 compared with the DMSO control. **F** The intracellular ATP content in U251 cells after treatment with different concentrations of bufalin (n = 3). *P* values were determined using one-way ANOVA. The relative expression value for each sample is shown along with the mean ± SD for each group. *p < 0.05, **p < 0.01 compared with DMSO control

We observed cell apoptosis induced by bufalin by co-staining the treated cells with MitoTracker Red CMX Ros, Annexin V-FITC and Hoechst 33,342 and detected apoptotic proteins by western blot analysis. We found that the phosphatidylserine of U251 cells everted from the inside of the plasma membrane to the cell surface, as indicated by staining with Annexin V-FITC. This result indicated that U251 cells undergo early apoptosis after exposure to bufalin for 12 h (Fig. 1A). Electron microscopy revealed numerous vacuolar-like structures in bufalin-treated cells, and the mitochondria, endoplasmic reticulum and other organelles were obviously damaged and scattered into fragments (Fig. 1B). The apoptosis-related protein results showed that bufalin can upregulate the expression of cytochrome C protein and the active form of caspase 3, cleaved caspase 3 (*p* < 0.05), to induce U251 cell apoptosis (Additional file 1: Fig. S2G).

The production of intracellular ROS and the consumption of GSH are two indicators of cellular oxidative stress. We detected the levels of ROS, GSH and ATP in U251 cells to determine the level of oxidative stress induced by bufalin. The results showed that the ROS levels in U251 cells treated with bufalin were increased, with a 4.93-fold increase in the ROS levels in 100 nM bufalin-treated cells compared with that of the control group (Fig. 1C and D), and the intracellular GSH/GSSG ratio and ATP content of bufalin-treated cells were significantly reduced, indicating that bufalin promotes GSH consumption and affects ATP production in U251 cells (Fig. 1E and F). Furthermore, we found that the amount of ROS NAC scavenged was increased by bufalin, and the CCK-8 results showed that pretreatment with 10 mM NAC for 1 h can partially inhibit the decrease in cell viability induced by bufalin (Additional file 1: Fig. S3 A–C).

### Bufalin induces DNA damage and arrests the cell cycle in the S phase

**Fig. 2:**
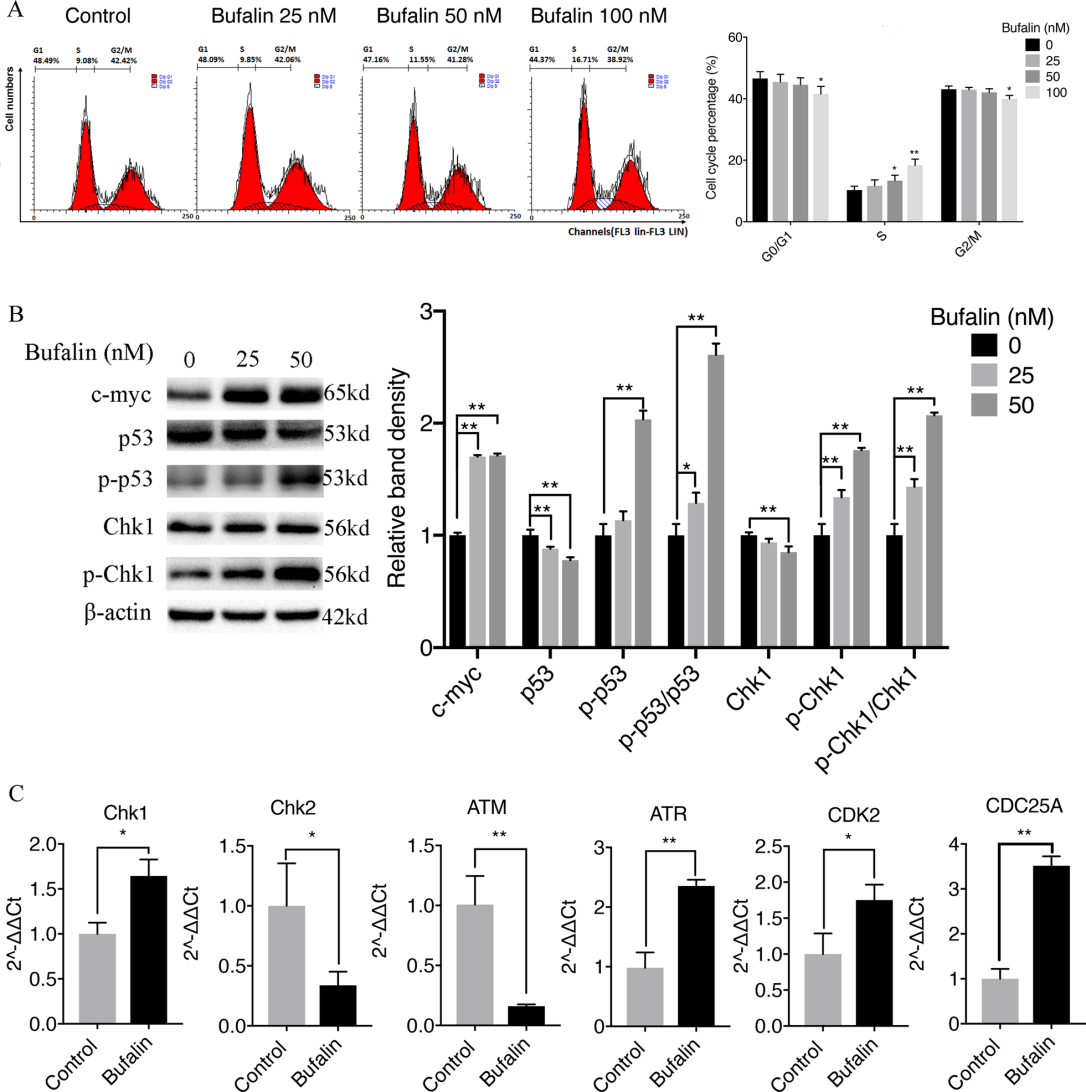
Bufalin (25, 50, and 100 nM) treatment for 24 h induces DNA damage and arrests the cell cycle in the S phase. **A** Proportion of cells in each phase of the cell cycle after bufalin treatment (n = 3). **B** Detection of DNA damage and cell cycle-related proteins by western blotting (n = 3). The Y axis shows the normalized values. **C** Detection of the mRNA levels of S-phase DNA damage-related genes (n = 3). The Y axis shows the normalized values. *P* values were determined using one-way ANOVA. The relative expression value for each sample is shown along with the mean ± SD for each group. *p < 0.05, **p < 0.01 compared with DMSO control

To assess whether the apoptosis of U251 cells caused by bufalin is related to cell cycle arrest, we performed flow cytometry assays to determine the percentage of U251 cells in each cycle after bufalin treatment. The results showed that bufalin treatment led to a concentration-dependent increase in the cellular population in the S phase, indicating that bufalin-induced U251 cell apoptosis is accompanied by arrest in the S phase (Fig. 2A).

An S-phase checkpoint protein senses whether the DNA is damaged and whether the damaged DNA molecule has been repaired to prevent damaged DNA from being replicated and passed down to daughter cells. Therefore, we used RT-QPCR to determine the mRNA levels of S-phase DNA damage-sensing molecules, namely, ATM, ATR, Chk1, Chk2, CDC25A and CDK2, in U251 cells treated with bufalin. The results indicated that the mRNA levels of Chk2 and ATM were significantly downregulated, and the mRNA expression levels of Chk1, ATR, CDC25A and CDK2 were significantly upregulated (*p* < 0.05 or *p* < 0.01) in U251 cells exposed to bufalin (Fig. 2C). In addition, the protein levels of p-Chk1 and p-p53 in U251 cells treated with bufalin for 24 h were significantly upregulated, and the expression levels of the Chk1 and p53 proteins were significantly downregulated, indicating that bufalin blocked U251 cell cycle progression in the S phase by regulating DNA damage-related genes (Fig. 2B).

### Bufalin induces mitochondrial dysfunction in U251 cells

**Fig. 3:**
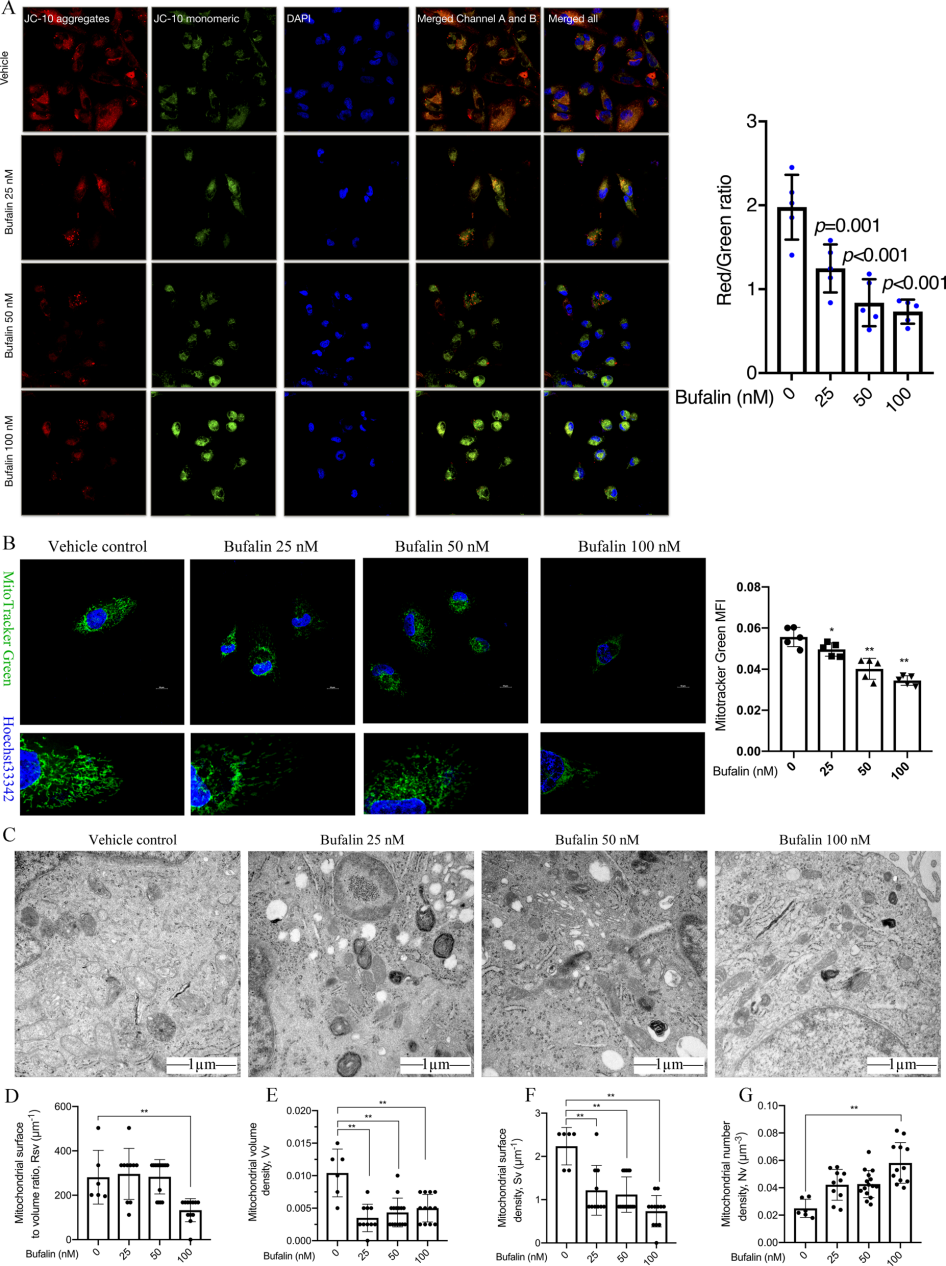
Bufalin (25, 50, and 100 nM) treatment for 24 h induces mitochondrial dysfunction in U251 cells. **A** Mitochondrial membrane potential in U251 cells as detected by JC-10 staining (n = 3). **B** Confocal microscopy observation of mitochondrial morphology and distribution as indicated by MitoTracker Green and Hoechst 33,342 staining (n = 5). The Y axis shows the intensity of green fluorescence. **C** Mitochondrial morphology of the U251 cells as observed with TEM. (D-G) Stereology analyses of Rsv, Vv, Sv and Nv in mitochondria. *P* values were determined using one-way ANOVA. The relative expression value or mitochondrial morphology data for each sample are shown along with the mean ± SD for each group. *p < 0.05, **p < 0.01 compared with DMSO control

Concentrated cellular JC-10 reversibly emits red fluorescence in cells with polarized mitochondrial membranes. When the mitochondrial membrane potential decreases and JC-10 cannot accumulate in the matrix of mitochondria, JC-10 is a monomer and emits green fluorescence. By calculating the ratio of red/green fluorescence, the MMP level in U251 cells was determined to evaluate the effect of bufalin on cell mitochondrial function. Following 6 h of treatment with bufalin, the intracellular MMP level decreased in U251 cells (Additional file 1: Fig. S4). After 12 h and 24 h of exposure to bufalin, the MMP was significantly reduced (Additional file 1: Fig. S4). Moreover, the MMP in the U251 cells treated with bufalin decreased with increases in the concentration administered (Fig. 3 A). These results indicate that bufalin can induce the loss of mitochondrial potential in a dose- and time-dependent manner.

MitoTracker green stain was used to evaluate the mitochondrial morphology and function of U251 cells after 24 h of bufalin treatment. The mitochondria in the untreated U251 cells were tightly distributed, and the network was clear, while the mitochondria in the bufalin-treated cells were scattered, the network was relatively loose, and the mitochondrial fluorescence was significantly less intense than in the control group (Fig. 3B).

TEM observation of the microscopic morphology of mitochondria of U251 cells revealed that bufalin can increase mitochondrial density in a cell, swell mitochondrial cristae, increase the number density of mitochondria (Nv), and reduce the surface volume ratio (Rsv), volume density (Vv) and mitochondrial surface density (Sv) of mitochondria, as shown in electron microscopic pictures (Fig. 3C–G). Considering these results, we suggest that bufalin can cause U251 cell mitochondria to split into a greater number of smaller mitochondria, thereby affecting mitochondrial function.

### Identification of bufalin targets by DARTS in U251 cells

**Fig. 4:**
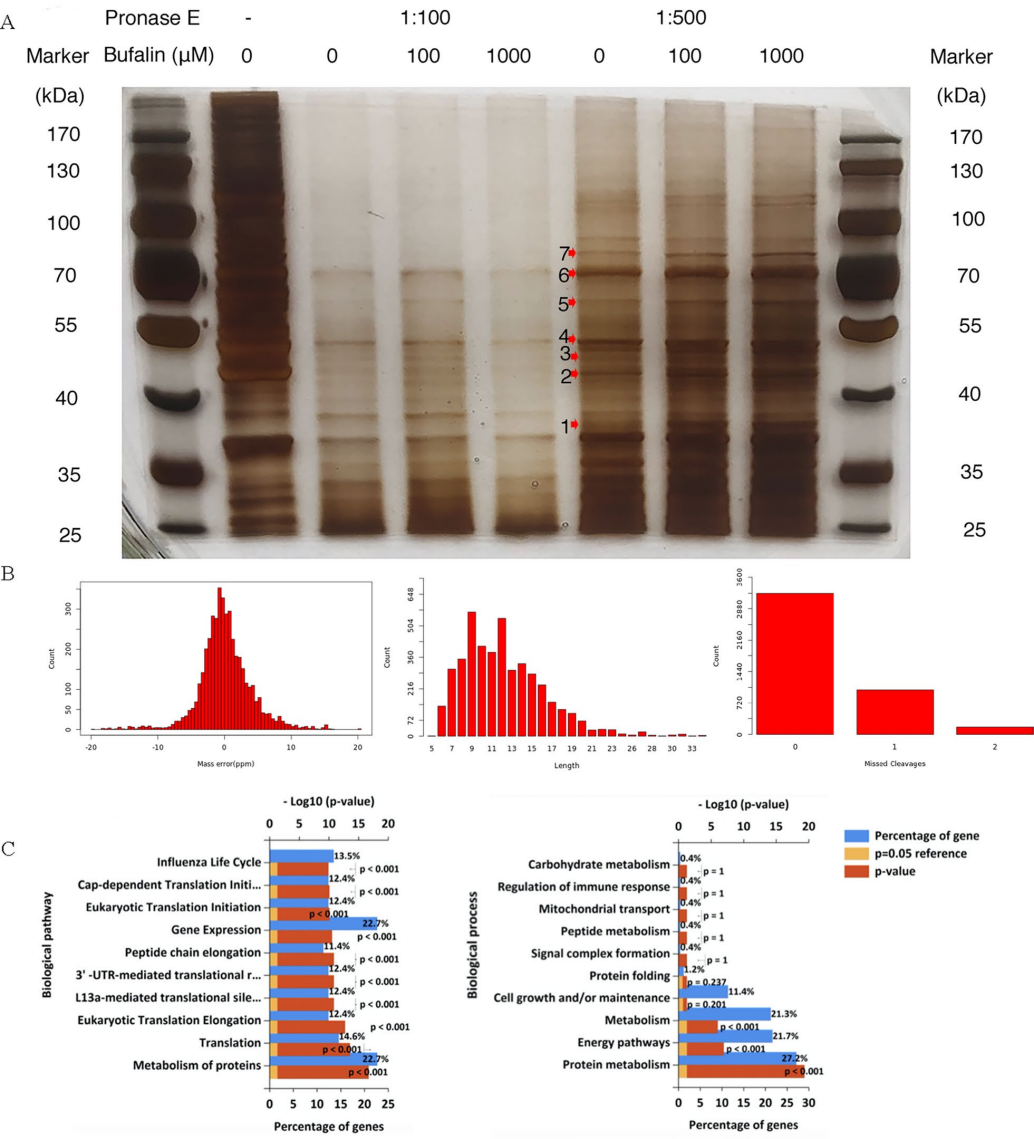
Target identification of bufalin acting on U251 cells. **A** The silver staining results of the DARTS-PAGE experiment. **B** The peptide length distribution, statistics indicating missing cleavage sites and the peptide mass deviation distribution. **C** GO function analysis of differential proteins

To clarify the target of bufalin acting on U251 cells, we silver-stained the SDS-PAGE gel in which the total protein samples from the U251 cells incubated overnight with bufalin were separated, and according to the silver staining gel cutting date of September 18, the bands with obvious differences were named 0918–1, 0918–2, …, 0918–7 based on the molecular weight from small to large (Fig. 4A). After decolorization, enzymolysis and extraction, the proteins were detected and analysed by a triple TOF 5600 LC–MS/MS system. The mass deviation for all peptides was less than 20 parts per million, and the mass spectrometry detection accuracy was good. Most peptides had intact cleavage sites, with a few peptides missing 1 or 2 cleavage sites, indicating that the digestion was sufficient and that the sample preparation met the standard (Fig. 4B). Based on the experience that the higher the protein abundance, the more spectra are collected, the proteins meeting the unused ≥ 1.3 criterion were eliminated, and a total of 258 differential proteins were obtained (Additional file 1: Table S2). A GO analysis of 258 differential proteins revealed that these proteins are mainly involved in protein metabolism, energy channels, metabolism, cell growth/maintenance, protein folding, mitochondrial transport and immune response regulation and other key pathways in the proliferation of glioma cells (Fig. 4C).

### Target screening and verification of bufalin action in U251 cells

**Fig. 5:**
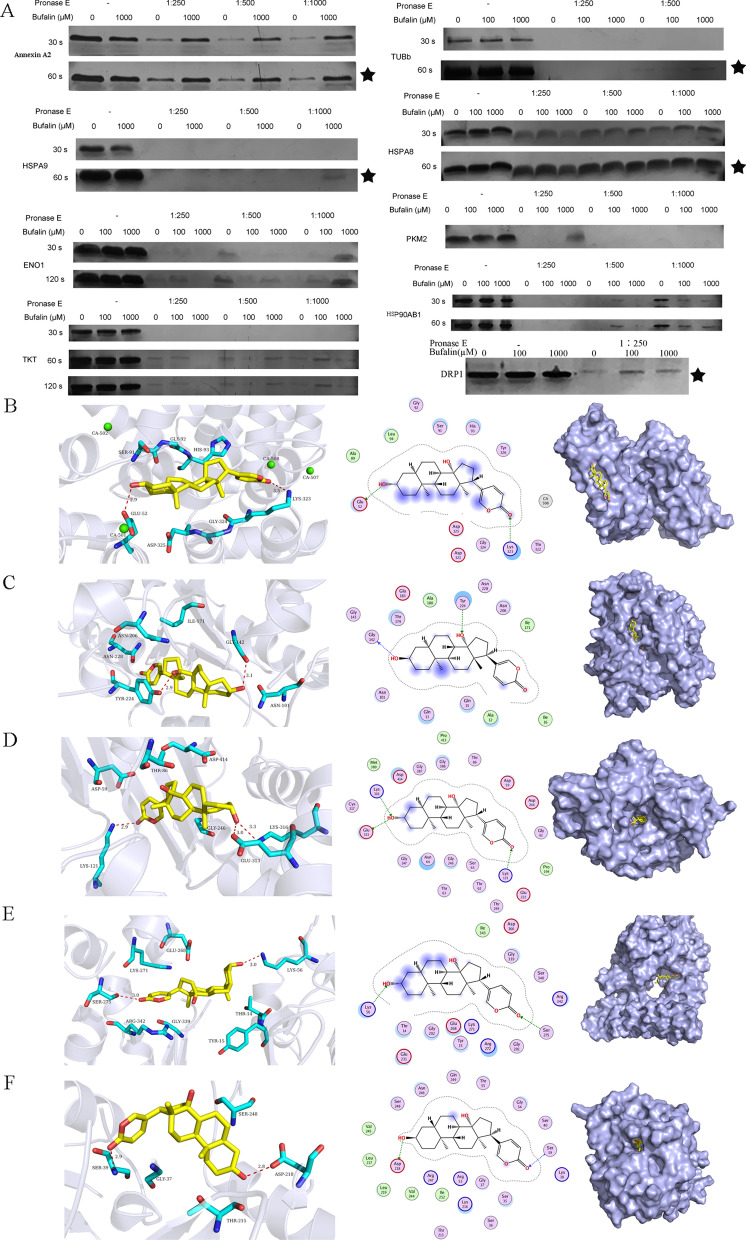
Target protein screening and verification of bufalin. **A** Silver staining verification upon pure protein and bufalin coincubation. ★ Probable target of bufalin after verification. 30 s and 60 s are the silver staining time. **B**–**F** 2D, 3D and surface binding models of bufalin with Annexin A2, TUBb, HSPA9, HSPA8, and DRP1 proteins

Differentially expressed proteins, including Annexin A2, TUBb, HSPA8, DRP1, HSPA9, PKM2, TKT, ENO1 and HSP90AB1 (Cloud-clone, Houston, America), were incubated with bufalin to verify their binding effect. The principle of DARTS technology is based on most of a drug binding to a target to make the target protein resistant to hydrolysis by the enzyme pronase E. From the results of the silver staining (Fig. 5A), we found that portions of the Annexin A2, DRP1, TUBb, HSPA8 and HSPA9 proteins were not enzymatically digested after bufalin incubation, compared with the control group that has not been incubated with bufalin. This indicates that the potential direct target proteins of bufalin in U251 cells are Annexin A2, DRP1, TUBb, HSPA8 and HSPA9 (Fig. 5A).

The results of molecular docking showed that the oxygen atom of the hydroxyl group in bufalin acts as a hydrogen bond, binding with the oxygen atom in the side chain of Glu52 in Annexin A2, the main chain oxygen atom of Gly142 in TUBb, the side chain nitrogen atom of Glu313 in HSPA9, and the side chain oxygen atom of Asp218 in DRP1 to form a hydrogen bond (Fig. 5B–D, F). The oxygen atom of the hydroxyl group in bufalin acts as a hydrogen bond acceptor, forming a hydrogen bond with the side chain oxygen atom of Tyr224 in TUBb, the side chain nitrogen atom of Ly316 in HSPA9, and the side chain oxygen atom of Lys56 in HSPA8 (Fig. 5C–E). The lactone oxygen atom in bufalin also acts as a hydrogen bond acceptor, combining with the nitrogen atom of the side chain of Lys323 in Annexin A2, the side chain nitrogen atom of Lys121 in HSPA9, the side chain of Ser275 in HSPA8 and the main chain nitrogen atom of Ser39 in DRP1 to form a hydrogen bond (Fig. 5 B–D, F). These results indicated that Annexin A2, DRP1, TUBb, HSPA8 and HSPA9 may be targets of bufalin in U251 cells.

### Bufalin binds directly to the Annexin A2 and DRP1 proteins

**Fig. 6:**
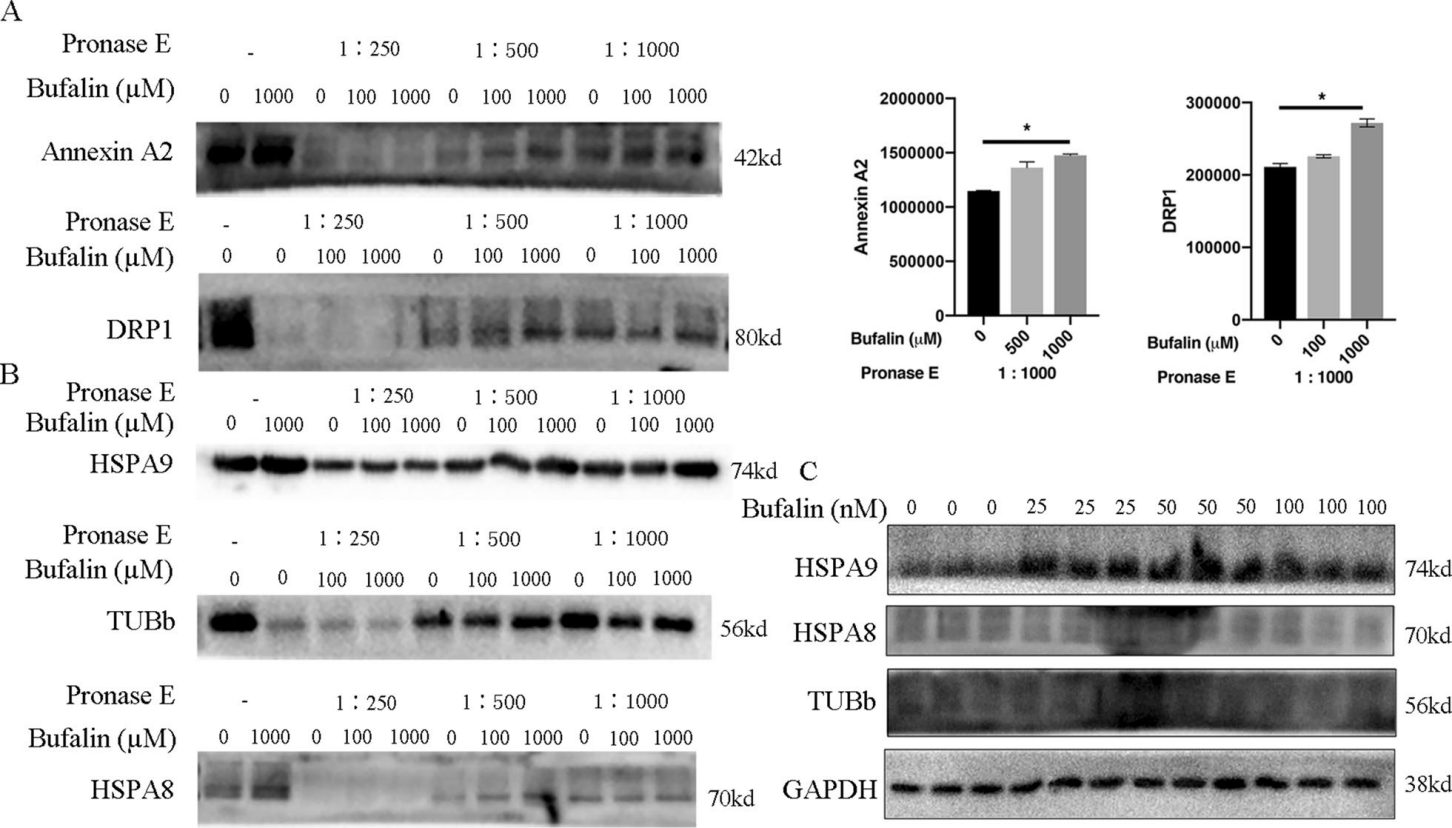
The direct target of bufalin. **A** The binding ability of bufalin with Annexin A2 and DRP1 in the total protein sample from U251 cells as verified by DARTS-western blot experiments. *P* values were determined using one-way ANOVA. The relative expression value for each sample is shown along with the mean ± SD for each group. *p < 0.05, compared with DMSO control (n = 3). **B** DARTS-western blot experiment was performed to detect the protein expression levels of HSPA8, HSPA9 and TUBb in pure protein samples of U251 cells after coincubation with bufalin (n = 3). **C** The protein expression levels of HSPA8, HSPA9 and TUBb in U251 cells after bufalin treatment as detected by western blot analysis (n = 3)

The total protein of U251 cells was incubated with bufalin and then enzymatically hydrolysed and denatured. DARTS-western blot experiments were performed to verify the direct binding ability of potential target proteins with bufalin. The results showed that the direct binding ability of Annexin A2 and DRP1 proteins was high and that these protein-bufalin compounds were not easily hydrolysed by protease (Fig. 6A), but HSPA9, TUBb and HSPA8 binding did not lead to the same results (Fig. 6B). Western blotting was performed to determine whether bufalin treatment for 24 h affects the protein expression of HSPA8, HSPA9 and/or TUBb in U251 cells. The results showed that, compared with that in the control group, the expression of HSPA9 protein increased significantly after the treatment with 25 nM or 50 nM bufalin for 24 h (*p* < 0.05), while there was no significant change in the 100 nM bufalin group (Fig. 6C). There was no change in HSPA8 and TUBb protein expression in either dose group. Considering these results, we suggest that Annexin A2 and DRP1 proteins are the direct target proteins of bufalin in U251 cells.

### Bufalin regulated Annexin A2 and DRP1 proteins to disrupt the mitochondrial division/fusion balance

**Fig. 7:**
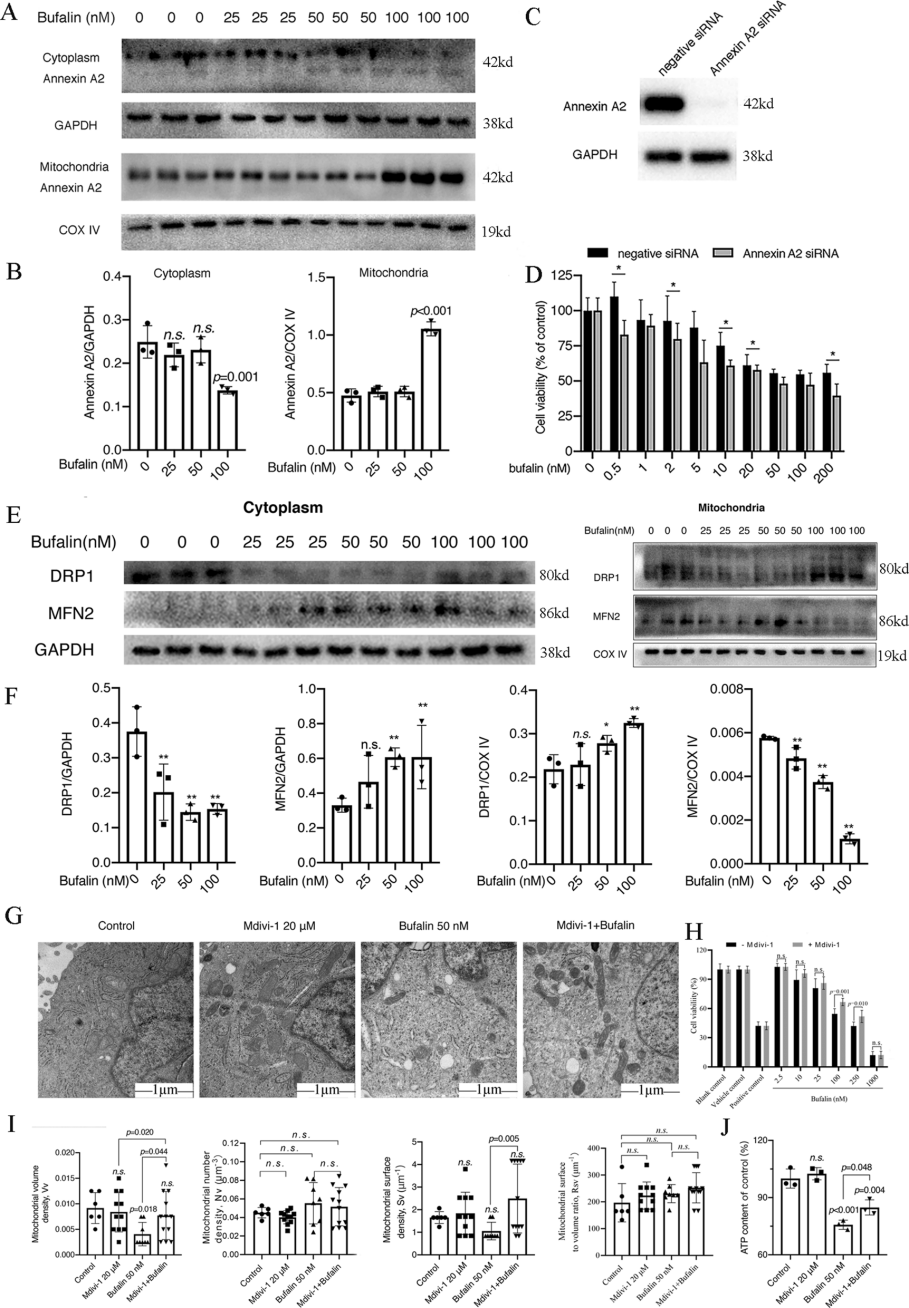
Bufalin induces U251 cell apoptosis by regulating Annexin A2 and DRP1 protein expression. **A** The expression of Annexin A2 protein in U251 cytoplasm and mitochondria after bufalin treatment for 24 h (n = 3). **B** Statistical analysis of Annexin A2 in U251 cytoplasm and mitochondria after bufalin treatment. **C** Verification of the Annexin A2 siRNA silencing effect. **D** The viability of U251 cells upon Annexin A2 silencing and bufalin treatment (n = 6). **E** The protein expression levels of DRP1 and MFN2 in the cytoplasm and mitochondria of U251 cells treated with bufalin for 24 h (n = 3). **F** Statistical analysis of DRP1 and MFN2 in U251 cytoplasm and mitochondria after bufalin treatment. **G** Mitochondrial morphology and structure of U251 cells were observed with TEM (n = 3). **H** Viability of U251 cells pretreated with Mdivi-1 (n = 6). **I** Stereology analyses the Rsv, Vv, Sv and Nv of mitochondria after pretreatment with Mdivi-1. **J** The intracellular ATP content of U251 cells (n = 3). *P* values were determined using one-way ANOVA. The relative expression value for each sample is shown along with the mean ± SD for each group. *p < 0.05, **p < 0.01 compared with DMSO control

To verify the role of Annexin A2 and DRP1 in bufalin-induced cell apoptosis, we examined the effect of Annexin A2 protein in bufalin-treated U251 cells for 24 h. The results showed that 100 nM bufalin significantly reduced the Annexin A2 protein content in the U251 cytoplasm and increased the Annexin A2 protein content in mitochondria (Fig. 7A and B). We therefore investigated whether Annexin A2 is related to bufalin-induced apoptosis of U251 cells. Further decrease in viability was observed in cells treated with bufalin after Annexin A2 was knocked down using siRNA (Fig. 7C and D).

The expression of the mitochondrial division-/fusion-related proteins DRP1 and MFN2 in U251 cells treated with different concentrations of bufalin was measured, and it was found that after bufalin treatment, the expression level of the DRP1 protein in the U251 cytoplasm was downregulated, but in mitochondria, it was upregulated (Fig. 7E and F). In contrast, the expression of the MFN2 protein was upregulated in the U251 cell cytoplasm but downregulated in mitochondria (Fig. 7E and F).

In addition, Mdivi-1 (a DRP1 inhibitor) attenuated the impact of bufalin on cell viability and mitochondrial structure changes. After pretreatment with 20 µM Mdivi-1, the viability of the Mdivi-1 + bufalin group cells was increased compared with that of the group without Mdivi-1 pretreatment (Fig. 7H). Mdivi-1 + bufalin treatment also slowed the bufalin-induced decrease in ATP content in U251 cells and protected mitochondrial function (Fig. 7J). The effect of 20 µM Mdivi-1 pretreatment on the changes in mitochondrial structure induced by bufalin was observed by electron microscopy. The Vv of the 50 nM bufalin group was significantly reduced, but there were no significant differences between Nv, Sv and Rsv, while the mitochondria in the U251 cells of the Mdivi-1 group did not change, indicating that 50 nM bufalin can change the structure of U251 cells (Fig. 7G and I). After pretreatment with 20 μM Mdivi-1, compared with the control group, the Nv, Vv, Rsv, and Sv in the 20 μM Mdivi-1 group did not change significantly, and compared with those in the 50 nM bufalin group, the Vv and Sv in the Mdivi-1 + bufalin group increased significantly; however, there was no significant change in the Nv or Rsv (Fig. 7G and I). In conclusion, these results indicated that bufalin can promote mitochondrial translocation of Annexin A2 and cause DRP1 oligomers to localize to the surface of mitochondria, disrupting the mitochondrial division/fusion balance and inducing U251 cell apoptosis.

## Discussion

Toad venom has been used throughout history for treating tumours and was recorded in the "Compendium of Materia Medica" to "cure all malignant swelling". It has been reported that bufalin, one of the main active compounds in toad venom, has a killing effect on a variety of tumour cells, and the combination of bufalin with 5-fluorouracil can attenuate the drug resistance of tumour cells, reduce their proliferation and induce their apoptosis [17, 18]. Previous studies showed that bufalin and related liposomes can induce the apoptosis of HepG2, HCT116, A549 and U251 cells in vitro and inhibit the growth of nude mouse xenograft gliomas in vivo [19, 20]. In the present study, the possible molecular mechanisms by which bufalin induces mitochondrial dysfunction to promote the apoptosis of glioma cells were elucidated.

This study showed that bufalin can significantly inhibit the proliferation and colony formation of U251 cells and cause structural loss of mitochondria and endoplasmic reticulum in these cells. The S-phase checkpoint is one of the important mechanisms of DNA damage repair, as this machinery senses whether the DNA of a cell is damaged and whether damaged DNA is repaired, thereby preventing damaged DNA from being replicated and passed down to daughter cells [21–23]. The results of our qRT-PCR experiments showed that the mRNA expression levels of Chk1, ATR, CDC25A and CDK2 in U251 cells treated with bufalin were significantly upregulated, and the ATR-Chk1-CDC25A-CDK2 pathway was activated to transmit DNA damage signals, which directly caused U251 cell cycle arrest in the S phase. The cytochrome C, caspase 3, c-myc, Chk1, p-Chk1, P53 and p-P53 protein expression levels in U251 cells were determined by western blotting, and the results were used to verify that apoptosis- and DNA damage-related proteins are induced by bufalin. The results showed that bufalin can upregulate cytochrome C expression, activate downstream caspase 3 protein phosphorylation, cause DNA damage, upregulate c-myc, and activate the p53 signalling pathway, causing cell cycle arrest in the S phase and inducing apoptosis.

The production of small amounts of oxygen free radicals in cells can maintain the balance of oxygen metabolism under the action of free radical scavenging enzymes or antioxidants under healthy conditions, but when excessive free radicals are generated by exogenous oxidants or the oxidation metabolism process exceed the capacity of the antioxidant system, and cells enter a state of oxidative stress [24]. A decrease in GSH content is a potential early activation signal for apoptosis, and the subsequent production of oxygen free radicals drives cells to enter apoptosis [25, 26]. We used DCFH-DA staining to determine the intracellular ROS content and the ratio of GSH/GSSG to evaluate the oxidative stress level of U251 cells after bufalin treatment. The results showed that bufalin can induce the overproduction of ROS in U251 cells, increase the consumption of GSH and promote redox imbalance in these cells. Na^+^/K^+^-ATPase is a protein embedded in the lipid bilayer of the plasma membrane of a cell, and it has with enzymatic activity and a carrier function [27, 28]. Na^+^/K^+^-ATPase can catalyse the hydrolysis of ATP to provide energy and can drive the transport of Na^+^ and K^+^ to both sides of the cell membrane, thus maintaining the potential on both sides of the cell membrane and playing an extremely important role in maintaining the normal physiological functions of cells [29, 30]. However, due to Na^+^/Ca^2+^ exchange, an imbalance in sodium and potassium ions is likely to disrupt intracellular calcium ion homeostasis [31]. By detecting the activity of Na^+^-K^+^-ATPase and the level of Ca^2+^ in U251 cells, we found that bufalin can disrupt the homeostasis of Na^+^, K^+^ and Ca^2+^ ions in U251 cells.

The imbalance of intracellular ions and the excessive generation of ROS trigger the opening of the mitochondrial permeability transition pore, and the expression of cytochrome C protein is upregulated, which activates the downstream caspase 3 protein, causing tumour cell mitochondrial dysfunction and inducing cell apoptosis [32]. Detection of cell mitochondrial membrane potential and intracellular ATP content is an important method for evaluating mitochondrial function [33]. MMP was measured by JC-10 staining, and cell ATP content was measured by an ATP content determination kit. Bufalin induced a significant decrease in ATP content and MMP levels in U251 cells, opened the mitochondrial permeability transition pore, and further induced mitochondrial dysfunction. Therefore, we believe that mitochondria play important roles in the tumour cell apoptosis induced by bufalin.

DARTS is a new technology based on the combination of small-molecule drugs and their target proteins, which leads to a decrease in the sensitivity of target proteins to protease degradation. Since the DARTS method does not require drug-protecting modification and is not dependent on drug activity, it can be widely used for drug screening and target identification. The enzymes used in the DARTS technology have been reported to include subtilisin, thermolysin and pronase. Subtilisin is functional only under alkaline conditions, which limits its use, and the use of small-molecule drugs increases the stability of nontarget proteins to which they bind ^35^. However, pronase has strong activity under neutral pH conditions and can specifically cleave folded or unfolded proteins, as well as the carboxyl side peptide chains of glutamic acid and aspartic acid in polypeptide chains. Therefore, pronase is often used as the enzyme in DARTS applications to identify targets of small-molecule drugs ^36^. In this study, DARTS-PAGE technology was employed to identify the target of bufalin in U251 cells. LC–MS/MS analysis was performed after enzymatic hydrolysis of the proteins in different silver-stained gel sections. Because of the high protein abundance, more spectra were collected. The number of reference spectra was used to represent the abundance of the reactive protein in the sample, and a total of 258 differential proteins were obtained. On this basis, protein molecular weight, peptides and number of spectra were combined to determine the representative differential proteins in the SDS-PAGE gel. The potential directly targeted proteins included Annexin A2, TUBb, HSPA8, DRP1 HSPA9, PKM2, TKT, ENO1 and HSP90AB1. Western blotting was used to detect the total protein in U251 cells incubated with bufalin, and Annexin A2 and DRP1 were found to be the direct target proteins of bufalin, while HSPA9, HSPA8 and TUBb proteins were less likely to be directly targeted by bufalin.

The Annexin A2 protein can enhance the activity of DNA polymerase and play an important role in DNA synthesis and cell proliferation [34, 35]. Its C-terminus can be combined with c-myc mRNA to regulate the function of the c-myc gene. The protein encoded by the c-myc gene can be used as a transcription factor to promote cell proliferation and plays multiple physiological and pathological roles in tumour formation [36]. Studies have reported that Annexin A2 protein is a substrate in the EGFR/ras/MAPK/PKC signaling pathway [37]. When EGFR and Annexin A2 proteins are simultaneously highly expressed in human glioma cells, the role of the MAPK signaling pathway is significantly enhanced, promoting the aggressive growth of human glioma cells [38]. We found that after treatment with 100 nM bufalin for 24 h, Annexin A2 protein expression in the cytoplasm of U251 cells was significantly downregulated, while Annexin A2 protein expression in mitochondria was significantly upregulated. Through siRNA technology, it was shown that after the expression level of Annexin A2 decreased, the cell survival rate of U251 cells was significantly reduced after bufalin treatment; that is, the sensitivity of U251 cells to bufalin was enhanced. These results indicate that bufalin treatment caused the Annexin A2 protein to undergo mitochondrial translocation, and Annexin A2 silencing reduced the activity of U251 cells treated with bufalin and promoted cell apoptosis. Moreover, to determine whether autophagy caused by bufalin treatment is related to the regulation of Annexin A2 that leads to cell apoptosis, further study is needed.

The mitochondrial division protein DRP1 is one of the key proteins that regulates mitochondrial division and fusion [39, 40]. The phosphorylation of DRP1 at Ser616 can promote mitochondrial division, and when DRP1 is phosphorylated at Ser637, mitochondrial division is inhibited [41]. Erk2, CaMKII, AMPK activation and Cdk1/cyclin B can promote the phosphorylation of DRP1 at Ser616 and increase the translocation of DRP1 to the mitochondrial surface [42]. FIS1 can promote DRP1 to enter mitochondria from the cytoplasm and then interact with DRP1 to promote mitochondrial fragmentation. Bax can migrate to the outer mitochondrial membrane to form a focal point and then interact with DRP1 and MFN2 to regulate mitochondrial morphology and apoptosis [43, 44]. Under normal conditions, the DRP1 protein is mainly distributed in the cytoplasm, and a small portion of DRP1 is distributed in the mitochondria. When mitochondria divide, DRP1 oligomers can wrap around the mitochondrial outer membrane and split it into membrane-related tubular structures [45]. Through a GTP hydrolysis-dependent mechanism, these tubular structures wrap around a cutting point, shrinking and cutting the mitochondrial membrane [46]. In addition, DRP1 plays an important role in mitochondrial mitosis and participates in the normal release of cytochrome C and the activation of caspase during apoptosis [47]. In the present study, we found that after bufalin treatment, the expression of mitochondrial division-/fusion-related proteins in U251 cells was abnormal, the DRP1 protein was translocated from the cytoplasm to mitochondria, and the MFN2 protein was released from mitochondria into the cytoplasm, disrupting the mitochondrial division/fusion balance in U251 cells. The DRP1 protein inhibitor Mdivi-1 partially ameliorated the abnormal structure of mitochondria caused by bufalin in U251 cells, protected mitochondrial function, and reduced the proportion of apoptotic cells. These results all suggest that the DRP1 protein is involved in bufalin-induced mitochondrial structure and functional abnormalities in U251 cells, which ultimately lead to cell apoptosis.

This study revealed a novel mechanism of bufalin function in cell apoptosis: the regulation of Annexin A2 and DRP1 proteins to induce mitochondrial dysfunction in U251 cells. The results provide a foundation for clinical application.

## Supplementary Information

**Additional file 1.** Additional figures and tables.

## Data Availability

The data and materials that support the current study are available from the corresponding author on reasonable request.

## Abbreviations

DARTS: Drug affinity responsive target stability
FCM: Flow cytometry
GSH: Glutathione
IC50: Inhibitory concentration
MMP: Mitochondrial membrane potential
NAC: *N*-acetylcysteine
Nv: Number density of mitochondria
ROS: Reactive oxygen species
Rsv: Surface volume ratio
Sv: Surface density
TEM: Transmission electron microscopy
and Vv: Volume density
