# Supplementary material for: Bufalin induces mitochondrial dysfunction and promotes apoptosis of glioma cells by regulating Annexin A2 and DRP1 protein expression
Source: Cancer Cell Int. 2021 Aug 10;21:424. doi: 10.1186/s12935-021-02137-x (PMC8353806; doi:10.1186/s12935-021-02137-x)
Supplement: Supplementary file 1 — Additional file 1. Additional figures and tables. [file 12935_2021_2137_MOESM1_ESM.doc]

**Bufalin induced mitochondrial dysfunction promotes apoptosis of glioma cells by regulating Annexin A2 and DRP1 proteins**

Yao Li ^1a^，Yan Zhang ^2a^，Xufang Wang^1^, Qian Yang^3^，Xuanxuan Zhou^3^，Junsheng Wu^1^，Xu Yang^1^，Yani Zhao^2^，Rui Lin^4^，Yanhua Xie^1^，Jiani Yuan^5*^，Xiaohui Zheng^1*^，Siwang Wang^1*^

1. Northwest University, Faculty of Life Science & Medicine, Key Laboratory Resource Biology & Biotechnology in Western China, Ministry of Education, Xi’an 710069, Shaanxi, China.
2. Department of acupuncture, Xi'an Hospital of Traditional Chinese Medicine, Xi'an 710021, Shaanxi, China
3. Department of Chinese Materia Medica and Natural Medicines, Air Force Medical University, Xi’an 710032 Shaanxi, China.
4. Department of Pharmacy, Xijing Hospital, Fourth Military Medical University, Xi’an 710032 Shaanxi, China.
5. Air Force Hospital of Western Theater Command, Chengdu 610083, Sichuan, China.

a These authors contributed to equally to this work.

***** Corresponding authors: **Jiani Yuan**, E-mail address: 419655632@qq.com; Phone number: 18200160975. **Xiaohui Zheng**, E-mail address: zhengxh318@nwu.edu.cn; Phone number: 13709265959. **Siwang Wang**, E-mail address: wangsiw@nwu.edu.cn; Phone number: 13909259880.

**Additional figures**


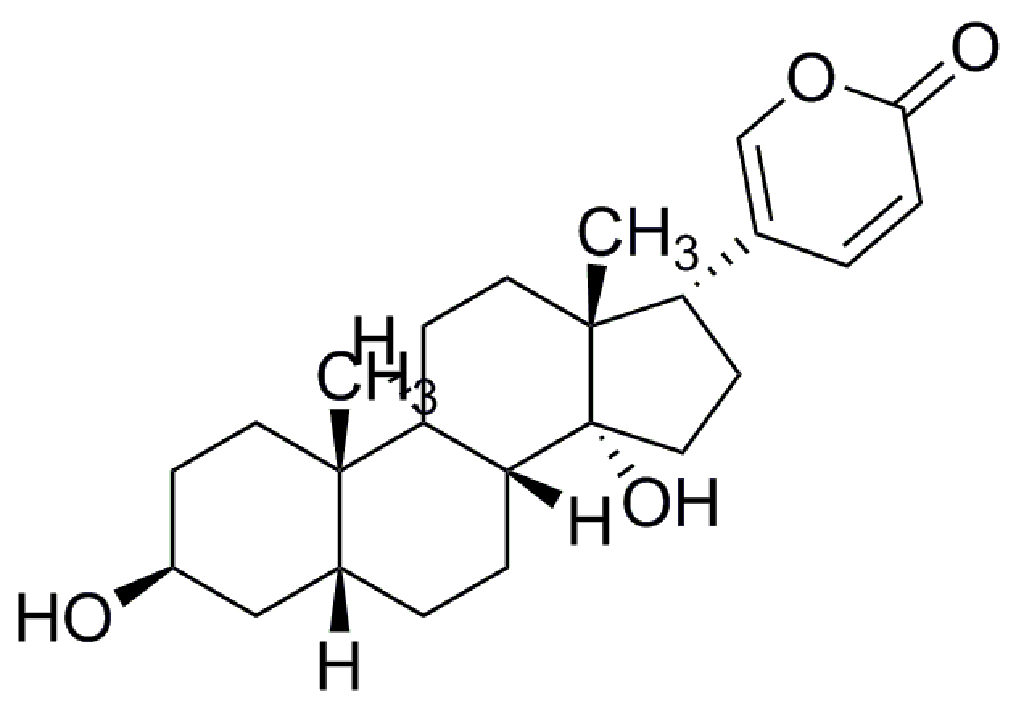


**Additional file 1: Fig. S1** The chemical structure of bufalin.


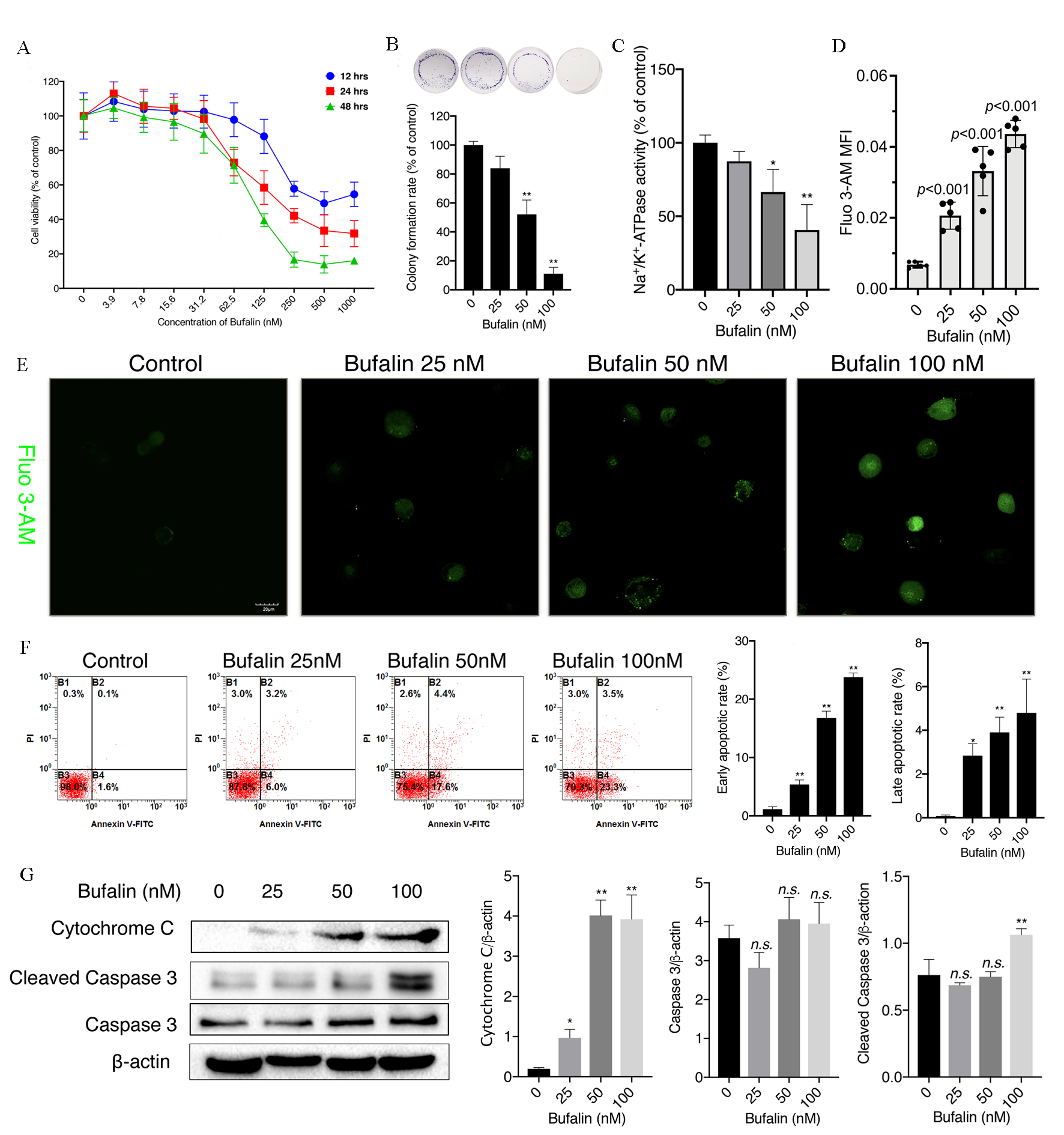


**Additional file 1: Fig. S2** Bufalin induces cell apoptosis. (A) Cell viability of U251 cells measured by CCK-8 (n=6). (B) Colony formation of U251 cells treated with bufalin (n=3). (C) Detection of Na^+^/K^+^-ATPase activity of U251 cells treated with bufalin (n=3). (D) The intracellular concentration of Ca^2+^. (E) Fluo3-AM staining to measure intracellular calcium ion levels of U251 cells (n=3). (F) Flow cytometry to detect the proportion of apoptotic cells after bufalin treated (n=3). (G) Detection of apoptosis-related proteins by western blot (n=3). *P* values were determined using one-way ANOVA. The relative expression value for each sample is shown along with mean ± SD for each group.**p* < 0.05, ***p* < 0.01 compared with DMSO control.


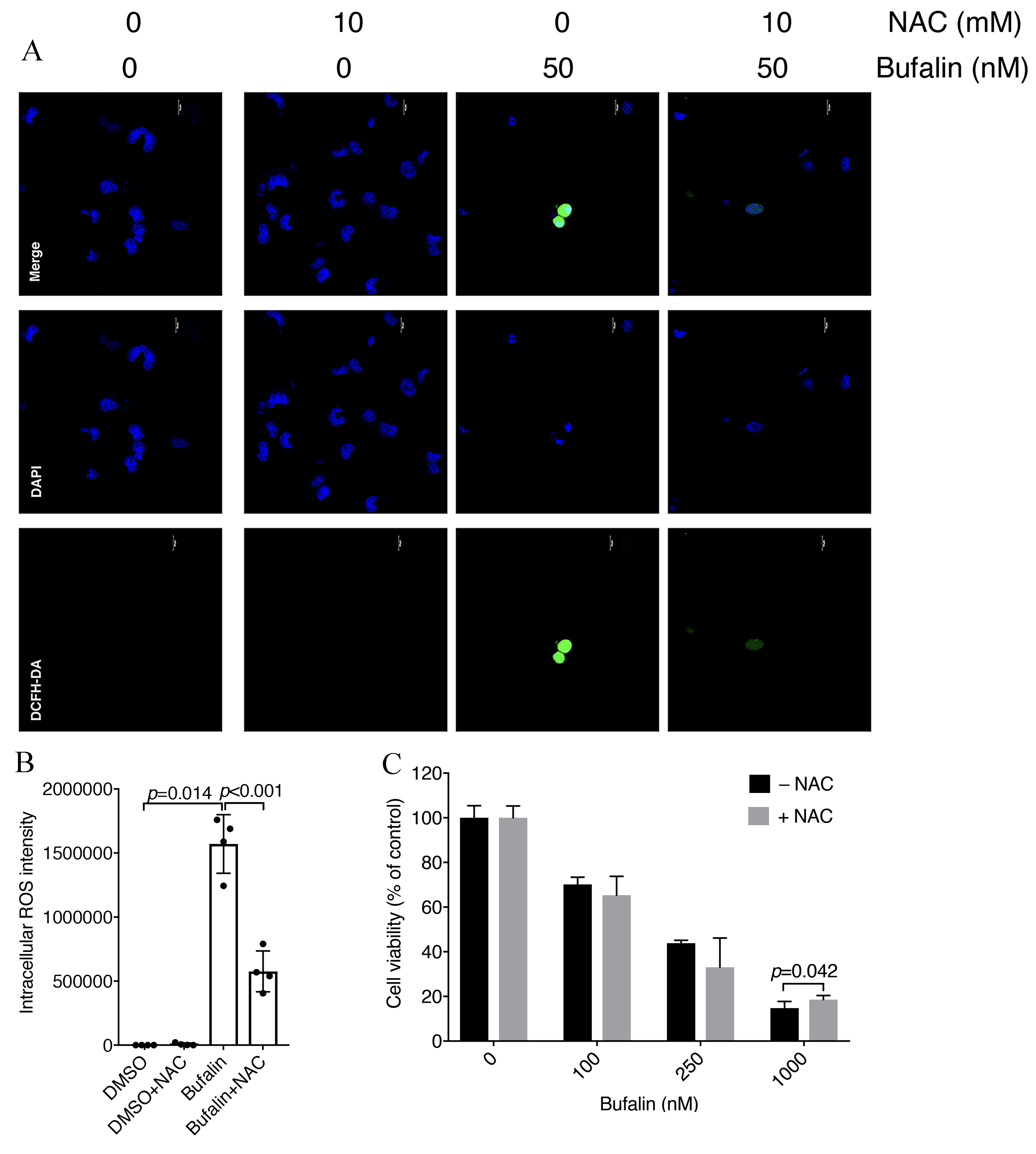


**Additional file 1: Fig. S3** Bufalin induces intracellular oxidative stress. (A-B) The intracellular ROS content of U251 cells after treated with bufalin and N-acetylcysteine (NAC). (C) The cell viability of U251 cells after treated with bufalin and N-acetylcysteine (NAC) (n=6). *P* values were determined using one-way ANOVA. The relative expression value for each sample is shown along with mean ± SD for each group.


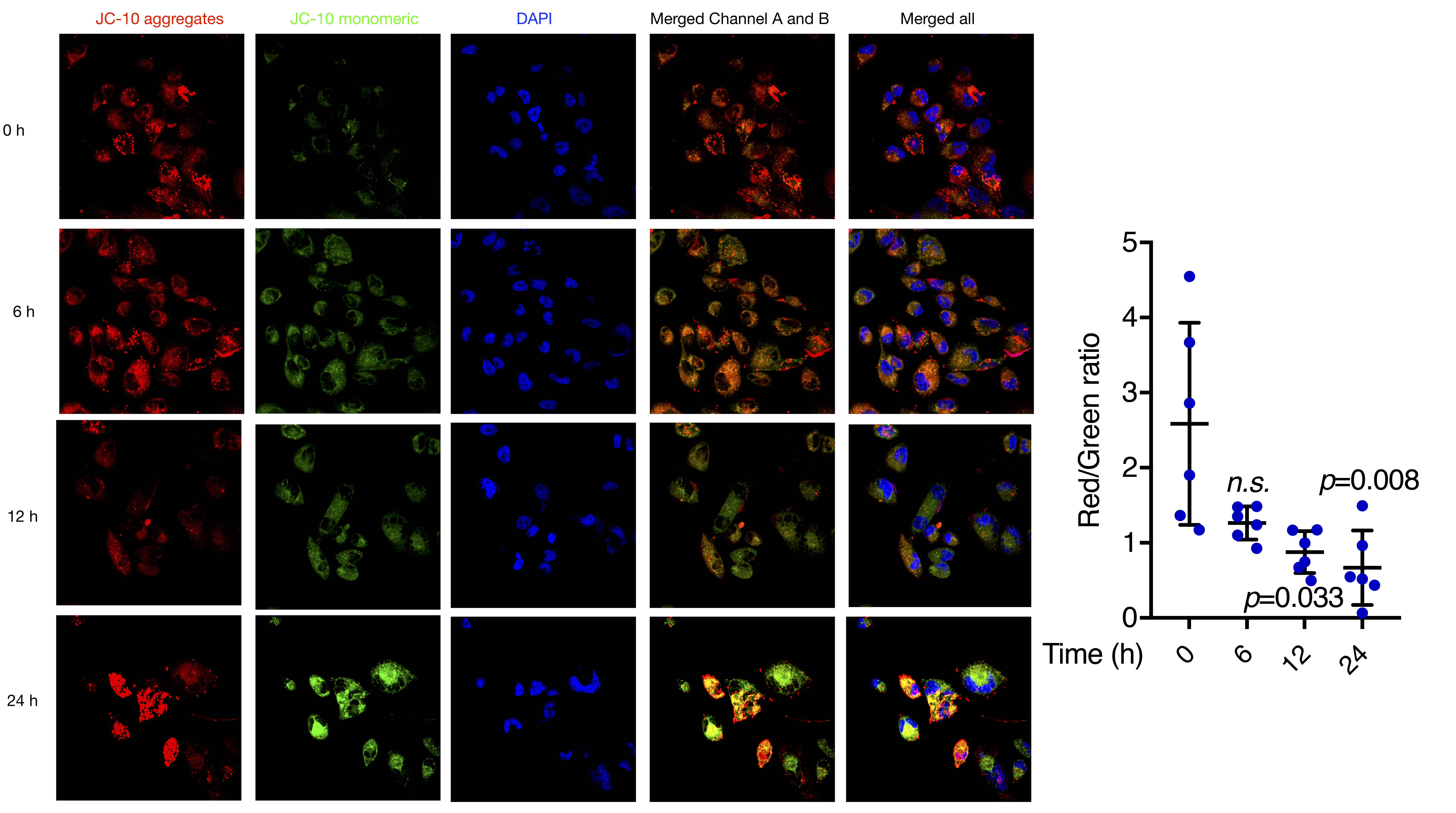


**Additional file 1: Fig. S4** Mitochondrial membrane potential in U251 cells treated with bufalin for different time (n=3). *P* values were determined using one-way ANOVA. The relative expression value for each sample is shown along with mean ± SD for each group.


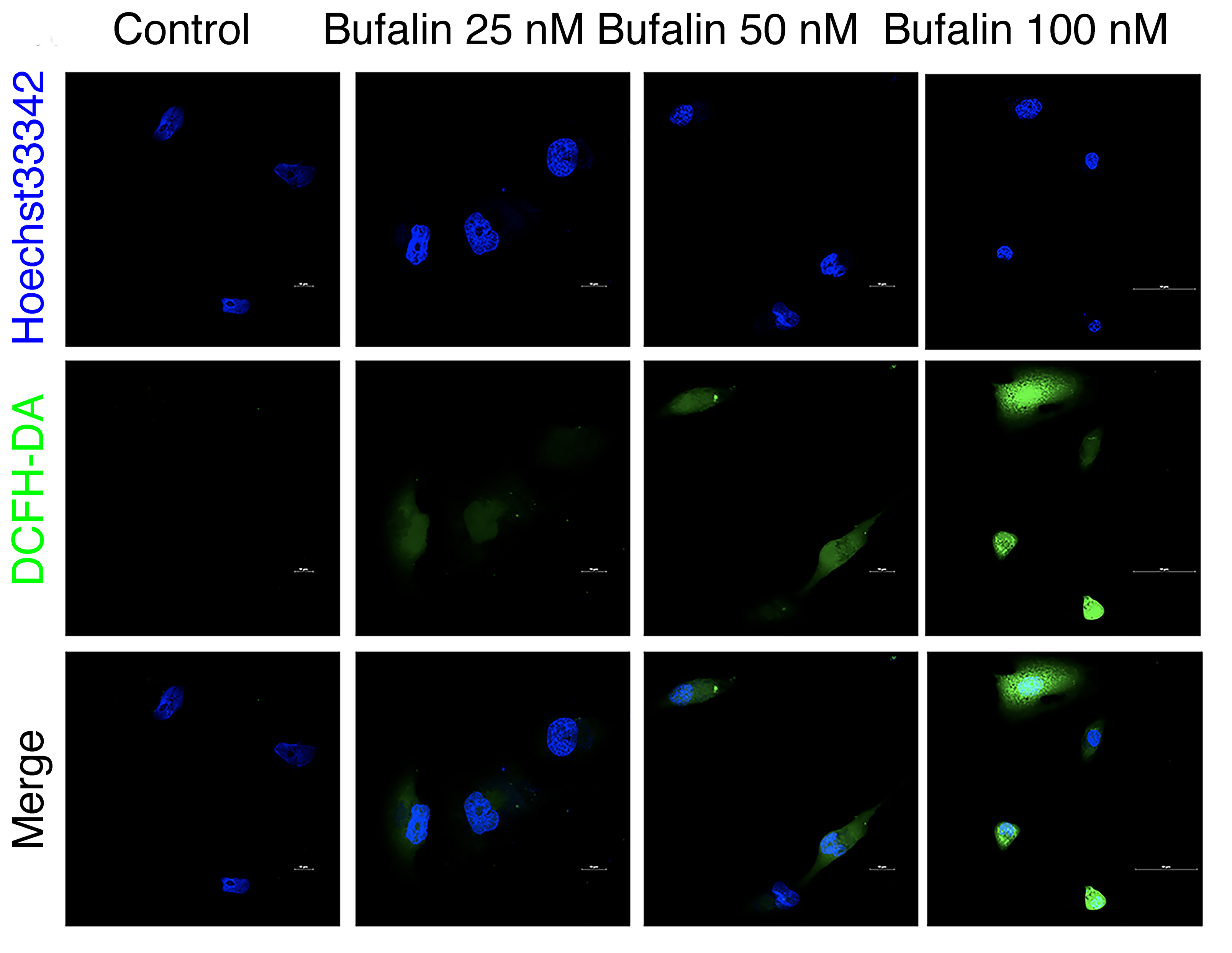


**Additional file 1: Fig. S5** The intracellular ROS content was observed by laser confocal microscope after DCFH-DA staining (n=3).

**Additional table**

**Additional file 1:** Table S1 Primers sequence information for qRT-PCR

| Gene | Sequence |
| --- | --- |
| *ATM-F* | GGCTATTCAGTGTGCGAGACA |
| *ATM-R* | TGGCTCCTTTCGGATGATGGA |
| *Chk2-F* | AACTCTTGGAAGTGGTGCCT |
| *Chk2-R* | GGTCTGCCTCTCTTGCTGAA |
| *ATR-F* | TCCCTTGAATACAGTGGCCTA |
| *ATR-R* | TCCTTGAAAGTACGGCAGTTC |
| *Chk1-F* | ATATGAAGCGTGCCGTAGACT |
| *Chk1-R* | TGCCTATGTCTGGCTCTATTCTG |
| *CDC25A-F* | CTCCTCCGAGTCAACAGATTCA |
| *CDC25A-R* | CAACAGCTTCTGAGGTAGGGA |
| *CDK2-F* | GTACCTCCCCTGGATGAAGAT |
| *CDK2-R* | CGAAATCCGCTTGTTAGGGTC |
| *β-actin-F:* | TGACGTGGACATCCGCAAAG |
| *β-actin-R:* | CTGGAAGGTGGACAGCGAGG |

**Additional file 1:** Table S2 Statistical table of differential protein detected by mass spectrum

| Accession | Gene | Description | Mw(kD) |
| --- | --- | --- | --- |
| E9PAV3 | NACA | Nascent polypeptide-associated complex subunit alpha, muscle-specific form OS=Homo sapiens OX=9606 GN=NACA PE=1 SV=1 | 205.422 |
| O00231 | PSMD11 | 26S proteasome non-ATPase regulatory subunit 11 OS=Homo sapiens OX=9606 GN=PSMD11 PE=1 SV=3 | 47.464 |
| O00571 | DDX3X | ATP-dependent RNA helicase DDX3X OS=Homo sapiens OX=9606 GN=DDX3X PE=1 SV=3 | 73.243 |
| O14787 | TNPO2 | Transportin-2 OS=Homo sapiens OX=9606 GN=TNPO2 PE=1 SV=3 | 101.388 |
| O14950 | MYL12B | Myosin regulatory light chain 12B OS=Homo sapiens OX=9606 GN=MYL12B PE=1 SV=2 | 19.779 |
| O14979 | HNRNPDL | Heterogeneous nuclear ribonucleoprotein D-like OS=Homo sapiens OX=9606 GN=HNRNPDL PE=1 SV=3 | 46.438 |
| O14980 | XPO1 | Exportin-1 OS=Homo sapiens OX=9606 GN=XPO1 PE=1 SV=1 | 123.386 |
| O15131 | KPNA5 | Importin subunit alpha-6 OS=Homo sapiens OX=9606 GN=KPNA5 PE=1 SV=2 | 60.349 |
| O43175 | PHGDH | D-3-phosphoglycerate dehydrogenase OS=Homo sapiens OX=9606 GN=PHGDH PE=1 SV=4 | 56.651 |
| O43707 | ACTN4 | Alpha-actinin-4 OS=Homo sapiens OX=9606 GN=ACTN4 PE=1 SV=2 | 104.854 |
| O43852 | CALU | Calumenin OS=Homo sapiens OX=9606 GN=CALU PE=1 SV=2 | 37.107 |
| O60506 | SYNCRIP | Heterogeneous nuclear ribonucleoprotein Q OS=Homo sapiens OX=9606 GN=SYNCRIP PE=1 SV=2 | 69.603 |
| O60568 | PLOD3 | Multifunctional procollagen lysine hydroxylase and glycosyltransferase LH3 OS=Homo sapiens OX=9606 GN=PLOD3 PE=1 SV=1 | 84.785 |
| O60814 | HIST1H2BK | Histone H2B type 1-K OS=Homo sapiens OX=9606 GN=HIST1H2BK PE=1 SV=3 | 13.89 |
| O75083 | WDR1 | WD repeat-containing protein 1 OS=Homo sapiens OX=9606 GN=WDR1 PE=1 SV=4 | 66.194 |
| O75131 | CPNE3 | Copine-3 OS=Homo sapiens OX=9606 GN=CPNE3 PE=1 SV=1 | 60.131 |
| O75369 | FLNB | Filamin-B OS=Homo sapiens OX=9606 GN=FLNB PE=1 SV=2 | 278.164 |
| O75390 | CS | Citrate synthase, mitochondrial OS=Homo sapiens OX=9606 GN=CS PE=1 SV=2 | 51.712 |
| O75718 | CRTAP | Cartilage-associated protein OS=Homo sapiens OX=9606 GN=CRTAP PE=1 SV=1 | 46.562 |
| O95757 | HSPA4L | Heat shock 70 kDa protein 4L OS=Homo sapiens OX=9606 GN=HSPA4L PE=1 SV=3 | 94.512 |
| O95782 | AP2A1 | AP-2 complex subunit alpha-1 OS=Homo sapiens OX=9606 GN=AP2A1 PE=1 SV=3 | 107.546 |
| P00338 | LDHA | L-lactate dehydrogenase A chain OS=Homo sapiens OX=9606 GN=LDHA PE=1 SV=2 | 36.689 |
| P00558 | PGK1 | Phosphoglycerate kinase 1 OS=Homo sapiens OX=9606 GN=PGK1 PE=1 SV=3 | 44.615 |
| P02042 | HBD | Hemoglobin subunit delta OS=Homo sapiens OX=9606 GN=HBD PE=1 SV=2 | 16.055 |
| P02545 | LMNA | Prelamin-A/C OS=Homo sapiens OX=9606 GN=LMNA PE=1 SV=1 | 74.139 |
| P02765 | AHSG | Alpha-2-HS-glycoprotein OS=Homo sapiens OX=9606 GN=AHSG PE=1 SV=2 | 39.341 |
| P02768 | ALB | Serum albumin OS=Homo sapiens OX=9606 GN=ALB PE=1 SV=2 | 69.367 |
| P02786 | TFRC | Transferrin receptor protein 1 OS=Homo sapiens OX=9606 GN=TFRC PE=1 SV=2 | 84.871 |
| P02788 | LTF | Lactotransferrin OS=Homo sapiens OX=9606 GN=LTF PE=1 SV=6 | 78.182 |
| O00429 | DNM1L（DRP1） | Dynamin relative protein 1 OS=Homo sapiens OX=9606 GN=DNM1L PE=1 SV=2 | 73.427 |
| P04075 | ALDOA | Fructose-bisphosphate aldolase A OS=Homo sapiens OX=9606 GN=ALDOA PE=1 SV=2 | 39.42 |
| P04083 | ANXA1 | Annexin A1 OS=Homo sapiens OX=9606 GN=ANXA1 PE=1 SV=2 | 38.714 |
| P04406 | GAPDH | Glyceraldehyde-3-phosphate dehydrogenase OS=Homo sapiens OX=9606 GN=GAPDH PE=1 SV=3 | 36.053 |
| P04745 | AMY1A | Alpha-amylase 1 OS=Homo sapiens OX=9606 GN=AMY1A PE=1 SV=2 | 57.768 |
| P04792 | HSPB1 | Heat shock protein beta-1 OS=Homo sapiens OX=9606 GN=HSPB1 PE=1 SV=2 | 22.783 |
| P04843 | RPN1 | Dolichyl-diphosphooligosaccharide--protein glycosyltransferase subunit 1 OS=Homo sapiens OX=9606 GN=RPN1 PE=1 SV=1 | 68.569 |
| P04908 | HIST1H2AB | Histone H2A type 1-B/E OS=Homo sapiens OX=9606 GN=HIST1H2AB PE=1 SV=2 | 14.135 |
| P05023 | ATP1A1 | Sodium/potassium-transporting ATPase subunit alpha-1 OS=Homo sapiens OX=9606 GN=ATP1A1 PE=1 SV=1 | 112.896 |
| P05121 | SERPINE1 | Plasminogen activator inhibitor 1 OS=Homo sapiens OX=9606 GN=SERPINE1 PE=1 SV=1 | 45.06 |
| P05141 | SLC25A5 | ADP/ATP translocase 2 OS=Homo sapiens OX=9606 GN=SLC25A5 PE=1 SV=7 | 32.852 |
| P05387 | RPLP2 | 60S acidic ribosomal protein P2 OS=Homo sapiens OX=9606 GN=RPLP2 PE=1 SV=1 | 11.665 |
| P05388 | RPLP0 | 60S acidic ribosomal protein P0 OS=Homo sapiens OX=9606 GN=RPLP0 PE=1 SV=1 | 34.274 |
| P06396 | GSN | Gelsolin OS=Homo sapiens OX=9606 GN=GSN PE=1 SV=1 | 85.698 |
| P06576 | ATP5F1B | ATP synthase subunit beta, mitochondrial OS=Homo sapiens OX=9606 GN=ATP5F1B PE=1 SV=3 | 56.56 |
| P06703 | S100A6 | Protein S100-A6 OS=Homo sapiens OX=9606 GN=S100A6 PE=1 SV=1 | 10.18 |
| P06733 | ENO1 | Alpha-enolase OS=Homo sapiens OX=9606 GN=ENO1 PE=1 SV=2 | 47.169 |
| P06744 | GPI | Glucose-6-phosphate isomerase OS=Homo sapiens OX=9606 GN=GPI PE=1 SV=4 | 63.147 |
| P07099 | EPHX1 | Epoxide hydrolase 1 OS=Homo sapiens OX=9606 GN=EPHX1 PE=1 SV=1 | 52.949 |
| P07195 | LDHB | L-lactate dehydrogenase B chain OS=Homo sapiens OX=9606 GN=LDHB PE=1 SV=2 | 36.638 |
| P07237 | P4HB | Protein disulfide-isomerase OS=Homo sapiens OX=9606 GN=P4HB PE=1 SV=3 | 57.116 |
| P07355 | ANXA2 | Annexin A2 OS=Homo sapiens OX=9606 GN=ANXA2 PE=1 SV=2 | 38.604 |
| P07384 | CAPN1 | Calpain-1 catalytic subunit OS=Homo sapiens OX=9606 GN=CAPN1 PE=1 SV=1 | 81.89 |
| P07437 | TUBB | Tubulin beta chain OS=Homo sapiens OX=9606 GN=TUBB PE=1 SV=2 | 49.671 |
| P07477 | PRSS1 | Trypsin-1 OS=Homo sapiens OX=9606 GN=PRSS1 PE=1 SV=1 | 26.558 |
| P07737 | PFN1 | Profilin-1 OS=Homo sapiens OX=9606 GN=PFN1 PE=1 SV=2 | 15.054 |
| P07900 | HSP90AA1 | Heat shock protein HSP 90-alpha OS=Homo sapiens OX=9606 GN=HSP90AA1 PE=1 SV=5 | 84.66 |
| P07910 | HNRNPC | Heterogeneous nuclear ribonucleoproteins C1/C2 OS=Homo sapiens OX=9606 GN=HNRNPC PE=1 SV=4 | 33.67 |
| P07954 | FH | Fumarate hydratase, mitochondrial OS=Homo sapiens OX=9606 GN=FH PE=1 SV=3 | 54.637 |
| P08133 | ANXA6 | Annexin A6 OS=Homo sapiens OX=9606 GN=ANXA6 PE=1 SV=3 | 75.873 |
| P08195 | SLC3A2 | 4F2 cell-surface antigen heavy chain OS=Homo sapiens OX=9606 GN=SLC3A2 PE=1 SV=3 | 67.994 |
| P08238 | HSP90AB1 | Heat shock protein HSP 90-beta OS=Homo sapiens OX=9606 GN=HSP90AB1 PE=1 SV=4 | 83.264 |
| P08670 | VIM | Vimentin OS=Homo sapiens OX=9606 GN=VIM PE=1 SV=4 | 53.652 |
| P08865 | RPSA | 40S ribosomal protein SA OS=Homo sapiens OX=9606 GN=RPSA PE=1 SV=4 | 32.854 |
| P09104 | ENO2 | Gamma-enolase OS=Homo sapiens OX=9606 GN=ENO2 PE=1 SV=3 | 47.269 |
| P0C0S8 | HIST1H2AG | Histone H2A type 1 OS=Homo sapiens OX=9606 GN=HIST1H2AG PE=1 SV=2 | 14.091 |
| P0CG47 | UBB | Polyubiquitin-B OS=Homo sapiens OX=9606 GN=UBB PE=1 SV=1 | 25.762 |
| P0DMV8 | HSPA1A | Heat shock 70 kDa protein 1A OS=Homo sapiens OX=9606 GN=HSPA1A PE=1 SV=1 | 70.052 |
| P10155 | RO60 | 60 kDa SS-A/Ro ribonucleoprotein OS=Homo sapiens OX=9606 GN=RO60 PE=1 SV=2 | 60.671 |
| P10412 | HIST1H1E | Histone H1.4 OS=Homo sapiens OX=9606 GN=HIST1H1E PE=1 SV=2 | 21.865 |
| P10809 | HSPD1 | 60 kDa heat shock protein, mitochondrial OS=Homo sapiens OX=9606 GN=HSPD1 PE=1 SV=2 | 61.055 |
| P11021 | HSPA5 | Endoplasmic reticulum chaperone BiP OS=Homo sapiens OX=9606 GN=HSPA5 PE=1 SV=2 | 72.333 |
| P11142 | HSPA8 | Heat shock cognate 71 kDa protein OS=Homo sapiens OX=9606 GN=HSPA8 PE=1 SV=1 | 70.898 |
| P11216 | PYGB | Glycogen phosphorylase, brain form OS=Homo sapiens OX=9606 GN=PYGB PE=1 SV=5 | 96.696 |
| P11413 | G6PD | Glucose-6-phosphate 1-dehydrogenase OS=Homo sapiens OX=9606 GN=G6PD PE=1 SV=4 | 59.257 |
| P11940 | PABPC1 | Polyadenylate-binding protein 1 OS=Homo sapiens OX=9606 GN=PABPC1 PE=1 SV=2 | 70.671 |
| P12081 | HARS | Histidine--tRNA ligase, cytoplasmic OS=Homo sapiens OX=9606 GN=HARS PE=1 SV=2 | 57.411 |
| P12277 | CKB | Creatine kinase B-type OS=Homo sapiens OX=9606 GN=CKB PE=1 SV=1 | 42.644 |
| P12814 | ACTN1 | Alpha-actinin-1 OS=Homo sapiens OX=9606 GN=ACTN1 PE=1 SV=2 | 103.058 |
| P12956 | XRCC6 | X-ray repair cross-complementing protein 6 OS=Homo sapiens OX=9606 GN=XRCC6 PE=1 SV=2 | 69.843 |
| P13639 | EEF2 | Elongation factor 2 OS=Homo sapiens OX=9606 GN=EEF2 PE=1 SV=4 | 95.338 |
| P13667 | PDIA4 | Protein disulfide-isomerase A4 OS=Homo sapiens OX=9606 GN=PDIA4 PE=1 SV=2 | 72.932 |
| P14136 | GFAP | Glial fibrillary acidic protein OS=Homo sapiens OX=9606 GN=GFAP PE=1 SV=1 | 49.88 |
| P14174 | MIF | Macrophage migration inhibitory factor OS=Homo sapiens OX=9606 GN=MIF PE=1 SV=4 | 12.476 |
| P14618 | PKM | Pyruvate kinase PKM OS=Homo sapiens OX=9606 GN=PKM PE=1 SV=4 | 57.937 |
| P14625 | HSP90B1 | Endoplasmin OS=Homo sapiens OX=9606 GN=HSP90B1 PE=1 SV=1 | 92.469 |
| P15121 | AKR1B1 | Aldo-keto reductase family 1 member B1 OS=Homo sapiens OX=9606 GN=AKR1B1 PE=1 SV=3 | 35.853 |
| P15311 | EZR | Ezrin OS=Homo sapiens OX=9606 GN=EZR PE=1 SV=4 | 69.413 |
| P16070 | CD44 | CD44 antigen OS=Homo sapiens OX=9606 GN=CD44 PE=1 SV=3 | 81.538 |
| P16930 | FAH | Fumarylacetoacetase OS=Homo sapiens OX=9606 GN=FAH PE=1 SV=2 | 46.374 |
| P17844 | DDX5 | Probable ATP-dependent RNA helicase DDX5 OS=Homo sapiens OX=9606 GN=DDX5 PE=1 SV=1 | 69.148 |
| P17987 | TCP1 | T-complex protein 1 subunit alpha OS=Homo sapiens OX=9606 GN=TCP1 PE=1 SV=1 | 60.344 |
| P18206 | VCL | Vinculin OS=Homo sapiens OX=9606 GN=VCL PE=1 SV=4 | 123.799 |
| P19338 | NCL | Nucleolin OS=Homo sapiens OX=9606 GN=NCL PE=1 SV=3 | 76.614 |
| P20073 | ANXA7 | Annexin A7 OS=Homo sapiens OX=9606 GN=ANXA7 PE=1 SV=3 | 52.739 |
| P20930 | FLG | Filaggrin OS=Homo sapiens OX=9606 GN=FLG PE=1 SV=3 | 435.17 |
| P21333 | FLNA | Filamin-A OS=Homo sapiens OX=9606 GN=FLNA PE=1 SV=4 | 280.739 |
| P22061 | PCMT1 | Protein-L-isoaspartate(D-aspartate) O-methyltransferase OS=Homo sapiens OX=9606 GN=PCMT1 PE=1 SV=4 | 24.636 |
| P22234 | PAICS | Multifunctional protein ADE2 OS=Homo sapiens OX=9606 GN=PAICS PE=1 SV=3 | 47.079 |
| P22314 | UBA1 | Ubiquitin-like modifier-activating enzyme 1 OS=Homo sapiens OX=9606 GN=UBA1 PE=1 SV=3 | 117.849 |
| P22626 | HNRNPA2B1 | Heterogeneous nuclear ribonucleoproteins A2/B1 OS=Homo sapiens OX=9606 GN=HNRNPA2B1 PE=1 SV=2 | 37.43 |
| P23526 | AHCY | Adenosylhomocysteinase OS=Homo sapiens OX=9606 GN=AHCY PE=1 SV=4 | 47.716 |
| P23528 | CFL1 | Cofilin-1 OS=Homo sapiens OX=9606 GN=CFL1 PE=1 SV=3 | 18.502 |
| P25398 | RPS12 | 40S ribosomal protein S12 OS=Homo sapiens OX=9606 GN=RPS12 PE=1 SV=3 | 14.515 |
| P25705 | ATP5F1A | ATP synthase subunit alpha, mitochondrial OS=Homo sapiens OX=9606 GN=ATP5F1A PE=1 SV=1 | 59.751 |
| P26006 | ITGA3 | Integrin alpha-3 OS=Homo sapiens OX=9606 GN=ITGA3 PE=1 SV=5 | 116.612 |
| P26038 | MSN | Moesin OS=Homo sapiens OX=9606 GN=MSN PE=1 SV=3 | 67.82 |
| P26196 | DDX6 | Probable ATP-dependent RNA helicase DDX6 OS=Homo sapiens OX=9606 GN=DDX6 PE=1 SV=2 | 54.417 |
| P26639 | TARS | Threonine--tRNA ligase, cytoplasmic OS=Homo sapiens OX=9606 GN=TARS PE=1 SV=3 | 83.435 |
| P26641 | EEF1G | Elongation factor 1-gamma OS=Homo sapiens OX=9606 GN=EEF1G PE=1 SV=3 | 50.119 |
| P27695 | APEX1 | DNA-(apurinic or apyrimidinic site) lyase OS=Homo sapiens OX=9606 GN=APEX1 PE=1 SV=2 | 35.555 |
| P27797 | CALR | Calreticulin OS=Homo sapiens OX=9606 GN=CALR PE=1 SV=1 | 48.142 |
| P29401 | TKT | Transketolase OS=Homo sapiens OX=9606 GN=TKT PE=1 SV=3 | 67.878 |
| P29692 | EEF1D | Elongation factor 1-delta OS=Homo sapiens OX=9606 GN=EEF1D PE=1 SV=5 | 31.122 |
| P30041 | PRDX6 | Peroxiredoxin-6 OS=Homo sapiens OX=9606 GN=PRDX6 PE=1 SV=3 | 25.035 |
| P30048 | PRDX3 | Thioredoxin-dependent peroxide reductase, mitochondrial OS=Homo sapiens OX=9606 GN=PRDX3 PE=1 SV=3 | 27.693 |
| P30101 | PDIA3 | Protein disulfide-isomerase A3 OS=Homo sapiens OX=9606 GN=PDIA3 PE=1 SV=4 | 56.782 |
| P30153 | PPP2R1A | Serine/threonine-protein phosphatase 2A 65 kDa regulatory subunit A alpha isoform OS=Homo sapiens OX=9606 GN=PPP2R1A PE=1 SV=4 | 65.309 |
| P31150 | GDI1 | Rab GDP dissociation inhibitor alpha OS=Homo sapiens OX=9606 GN=GDI1 PE=1 SV=2 | 50.583 |
| P31153 | MAT2A | S-adenosylmethionine synthase isoform type-2 OS=Homo sapiens OX=9606 GN=MAT2A PE=1 SV=1 | 43.661 |
| P31689 | DNAJA1 | DnaJ homolog subfamily A member 1 OS=Homo sapiens OX=9606 GN=DNAJA1 PE=1 SV=2 | 44.868 |
| P31942 | HNRNPH3 | Heterogeneous nuclear ribonucleoprotein H3 OS=Homo sapiens OX=9606 GN=HNRNPH3 PE=1 SV=2 | 36.926 |
| P31943 | HNRNPH1 | Heterogeneous nuclear ribonucleoprotein H OS=Homo sapiens OX=9606 GN=HNRNPH1 PE=1 SV=4 | 49.229 |
| P31948 | STIP1 | Stress-induced-phosphoprotein 1 OS=Homo sapiens OX=9606 GN=STIP1 PE=1 SV=1 | 62.639 |
| P34932 | HSPA4 | Heat shock 70 kDa protein 4 OS=Homo sapiens OX=9606 GN=HSPA4 PE=1 SV=4 | 94.331 |
| P35237 | SERPINB6 | Serpin B6 OS=Homo sapiens OX=9606 GN=SERPINB6 PE=1 SV=3 | 42.622 |
| P35268 | RPL22 | 60S ribosomal protein L22 OS=Homo sapiens OX=9606 GN=RPL22 PE=1 SV=2 | 14.787 |
| P35579 | MYH9 | Myosin-9 OS=Homo sapiens OX=9606 GN=MYH9 PE=1 SV=4 | 226.532 |
| P35606 | COPB2 | Coatomer subunit beta' OS=Homo sapiens OX=9606 GN=COPB2 PE=1 SV=2 | 102.487 |
| P35998 | PSMC2 | 26S proteasome regulatory subunit 7 OS=Homo sapiens OX=9606 GN=PSMC2 PE=1 SV=3 | 48.634 |
| P36578 | RPL4 | 60S ribosomal protein L4 OS=Homo sapiens OX=9606 GN=RPL4 PE=1 SV=5 | 47.697 |
| P36873 | PPP1CC | Serine/threonine-protein phosphatase PP1-gamma catalytic subunit OS=Homo sapiens OX=9606 GN=PPP1CC PE=1 SV=1 | 36.984 |
| P37802 | TAGLN2 | Transgelin-2 OS=Homo sapiens OX=9606 GN=TAGLN2 PE=1 SV=3 | 22.391 |
| P38646 | HSPA9 | Stress-70 protein, mitochondrial OS=Homo sapiens OX=9606 GN=HSPA9 PE=1 SV=2 | 73.681 |
| P39023 | RPL3 | 60S ribosomal protein L3 OS=Homo sapiens OX=9606 GN=RPL3 PE=1 SV=2 | 46.109 |
| P39656 | DDOST | Dolichyl-diphosphooligosaccharide--protein glycosyltransferase 48 kDa subunit OS=Homo sapiens OX=9606 GN=DDOST PE=1 SV=4 | 50.801 |
| P40227 | CCT6A | T-complex protein 1 subunit zeta OS=Homo sapiens OX=9606 GN=CCT6A PE=1 SV=3 | 58.024 |
| P40925 | MDH1 | Malate dehydrogenase, cytoplasmic OS=Homo sapiens OX=9606 GN=MDH1 PE=1 SV=4 | 36.426 |
| P40926 | MDH2 | Malate dehydrogenase, mitochondrial OS=Homo sapiens OX=9606 GN=MDH2 PE=1 SV=3 | 35.503 |
| P41250 | GARS | Glycine--tRNA ligase OS=Homo sapiens OX=9606 GN=GARS PE=1 SV=3 | 83.166 |
| P43034 | PAFAH1B1 | Platelet-activating factor acetylhydrolase IB subunit alpha OS=Homo sapiens OX=9606 GN=PAFAH1B1 PE=1 SV=2 | 46.638 |
| P43304 | GPD2 | Glycerol-3-phosphate dehydrogenase, mitochondrial OS=Homo sapiens OX=9606 GN=GPD2 PE=1 SV=3 | 80.853 |
| P46783 | RPS10 | 40S ribosomal protein S10 OS=Homo sapiens OX=9606 GN=RPS10 PE=1 SV=1 | 18.898 |
| P46940 | IQGAP1 | Ras GTPase-activating-like protein IQGAP1 OS=Homo sapiens OX=9606 GN=IQGAP1 PE=1 SV=1 | 189.252 |
| P47897 | QARS | Glutamine--tRNA ligase OS=Homo sapiens OX=9606 GN=QARS PE=1 SV=1 | 87.799 |
| P47914 | RPL29 | 60S ribosomal protein L29 OS=Homo sapiens OX=9606 GN=RPL29 PE=1 SV=2 | 17.752 |
| P48147 | PREP | Prolyl endopeptidase OS=Homo sapiens OX=9606 GN=PREP PE=1 SV=2 | 80.7 |
| P48643 | CCT5 | T-complex protein 1 subunit epsilon OS=Homo sapiens OX=9606 GN=CCT5 PE=1 SV=1 | 59.671 |
| P49327 | FASN | Fatty acid synthase OS=Homo sapiens OX=9606 GN=FASN PE=1 SV=3 | 273.427 |
| P49748 | ACADVL | Very long-chain specific acyl-CoA dehydrogenase, mitochondrial OS=Homo sapiens OX=9606 GN=ACADVL PE=1 SV=1 | 70.39 |
| P50395 | GDI2 | Rab GDP dissociation inhibitor beta OS=Homo sapiens OX=9606 GN=GDI2 PE=1 SV=2 | 50.663 |
| P50416 | CPT1A | Carnitine O-palmitoyltransferase 1, liver isoform OS=Homo sapiens OX=9606 GN=CPT1A PE=1 SV=2 | 88.368 |
| P50454 | SERPINH1 | Serpin H1 OS=Homo sapiens OX=9606 GN=SERPINH1 PE=1 SV=2 | 46.441 |
| P50502 | ST13 | Hsc70-interacting protein OS=Homo sapiens OX=9606 GN=ST13 PE=1 SV=2 | 41.332 |
| P50991 | CCT4 | T-complex protein 1 subunit delta OS=Homo sapiens OX=9606 GN=CCT4 PE=1 SV=4 | 57.924 |
| P50995 | ANXA11 | Annexin A11 OS=Homo sapiens OX=9606 GN=ANXA11 PE=1 SV=1 | 54.39 |
| P51659 | HSD17B4 | Peroxisomal multifunctional enzyme type 2 OS=Homo sapiens OX=9606 GN=HSD17B4 PE=1 SV=3 | 79.686 |
| P52209 | PGD | 6-phosphogluconate dehydrogenase, decarboxylating OS=Homo sapiens OX=9606 GN=PGD PE=1 SV=3 | 53.14 |
| P52272 | HNRNPM | Heterogeneous nuclear ribonucleoprotein M OS=Homo sapiens OX=9606 GN=HNRNPM PE=1 SV=3 | 77.516 |
| P52292 | KPNA2 | Importin subunit alpha-1 OS=Homo sapiens OX=9606 GN=KPNA2 PE=1 SV=1 | 57.862 |
| P52597 | HNRNPF | Heterogeneous nuclear ribonucleoprotein F OS=Homo sapiens OX=9606 GN=HNRNPF PE=1 SV=3 | 45.672 |
| P52907 | CAPZA1 | F-actin-capping protein subunit alpha-1 OS=Homo sapiens OX=9606 GN=CAPZA1 PE=1 SV=3 | 32.923 |
| P53396 | ACLY | ATP-citrate synthase OS=Homo sapiens OX=9606 GN=ACLY PE=1 SV=3 | 120.839 |
| P53621 | COPA | Coatomer subunit alpha OS=Homo sapiens OX=9606 GN=COPA PE=1 SV=2 | 138.346 |
| P53992 | SEC24C | Protein transport protein Sec24C OS=Homo sapiens OX=9606 GN=SEC24C PE=1 SV=3 | 118.325 |
| P54136 | RARS | Arginine--tRNA ligase, cytoplasmic OS=Homo sapiens OX=9606 GN=RARS PE=1 SV=2 | 75.379 |
| P55072 | VCP | Transitional endoplasmic reticulum ATPase OS=Homo sapiens OX=9606 GN=VCP PE=1 SV=4 | 89.322 |
| P55884 | EIF3B | Eukaryotic translation initiation factor 3 subunit B OS=Homo sapiens OX=9606 GN=EIF3B PE=1 SV=3 | 92.482 |
| P60174 | TPI1 | Triosephosphate isomerase OS=Homo sapiens OX=9606 GN=TPI1 PE=1 SV=3 | 30.791 |
| P60228 | EIF3E | Eukaryotic translation initiation factor 3 subunit E OS=Homo sapiens OX=9606 GN=EIF3E PE=1 SV=1 | 52.221 |
| P60709 | ACTB | Actin, cytoplasmic 1 OS=Homo sapiens OX=9606 GN=ACTB PE=1 SV=1 | 41.737 |
| P60842 | EIF4A1 | Eukaryotic initiation factor 4A-I OS=Homo sapiens OX=9606 GN=EIF4A1 PE=1 SV=1 | 46.154 |
| P61158 | ACTR3 | Actin-related protein 3 OS=Homo sapiens OX=9606 GN=ACTR3 PE=1 SV=3 | 47.371 |
| P61163 | ACTR1A | Alpha-centractin OS=Homo sapiens OX=9606 GN=ACTR1A PE=1 SV=1 | 42.614 |
| P61221 | ABCE1 | ATP-binding cassette sub-family E member 1 OS=Homo sapiens OX=9606 GN=ABCE1 PE=1 SV=1 | 67.314 |
| P61978 | HNRNPK | Heterogeneous nuclear ribonucleoprotein K OS=Homo sapiens OX=9606 GN=HNRNPK PE=1 SV=1 | 50.976 |
| P62241 | RPS8 | 40S ribosomal protein S8 OS=Homo sapiens OX=9606 GN=RPS8 PE=1 SV=2 | 24.205 |
| P62249 | RPS16 | 40S ribosomal protein S16 OS=Homo sapiens OX=9606 GN=RPS16 PE=1 SV=2 | 16.445 |
| P62258 | YWHAE | 14-3-3 protein epsilon OS=Homo sapiens OX=9606 GN=YWHAE PE=1 SV=1 | 29.174 |
| P62333 | PSMC6 | 26S proteasome regulatory subunit 10B OS=Homo sapiens OX=9606 GN=PSMC6 PE=1 SV=1 | 44.173 |
| P62701 | RPS4X | 40S ribosomal protein S4, X isoform OS=Homo sapiens OX=9606 GN=RPS4X PE=1 SV=2 | 29.598 |
| P62805 | HIST1H4A | Histone H4 OS=Homo sapiens OX=9606 GN=HIST1H4A PE=1 SV=2 | 11.367 |
| P62847 | RPS24 | 40S ribosomal protein S24 OS=Homo sapiens OX=9606 GN=RPS24 PE=1 SV=1 | 15.423 |
| P62888 | RPL30 | 60S ribosomal protein L30 OS=Homo sapiens OX=9606 GN=RPL30 PE=1 SV=2 | 12.784 |
| P62899 | RPL31 | 60S ribosomal protein L31 OS=Homo sapiens OX=9606 GN=RPL31 PE=1 SV=1 | 14.463 |
| P62917 | RPL8 | 60S ribosomal protein L8 OS=Homo sapiens OX=9606 GN=RPL8 PE=1 SV=2 | 28.025 |
| P62937 | PPIA | Peptidyl-prolyl cis-trans isomerase A OS=Homo sapiens OX=9606 GN=PPIA PE=1 SV=2 | 18.012 |
| P63220 | RPS21 | 40S ribosomal protein S21 OS=Homo sapiens OX=9606 GN=RPS21 PE=1 SV=1 | 9.111 |
| P63241 | EIF5A | Eukaryotic translation initiation factor 5A-1 OS=Homo sapiens OX=9606 GN=EIF5A PE=1 SV=2 | 16.832 |
| P67809 | YBX1 | Nuclease-sensitive element-binding protein 1 OS=Homo sapiens OX=9606 GN=YBX1 PE=1 SV=3 | 35.924 |
| P68104 | EEF1A1 | Elongation factor 1-alpha 1 OS=Homo sapiens OX=9606 GN=EEF1A1 PE=1 SV=1 | 50.141 |
| P68363 | TUBA1B | Tubulin alpha-1B chain OS=Homo sapiens OX=9606 GN=TUBA1B PE=1 SV=1 | 50.152 |
| P68371 | TUBB4B | Tubulin beta-4B chain OS=Homo sapiens OX=9606 GN=TUBB4B PE=1 SV=1 | 49.831 |
| P68431 | HIST1H3A | Histone H3.1 OS=Homo sapiens OX=9606 GN=HIST1H3A PE=1 SV=2 | 15.404 |
| P78371 | CCT2 | T-complex protein 1 subunit beta OS=Homo sapiens OX=9606 GN=CCT2 PE=1 SV=4 | 57.488 |
| P81605 | DCD | Dermcidin OS=Homo sapiens OX=9606 GN=DCD PE=1 SV=2 | 11.284 |
| Q00325 | SLC25A3 | Phosphate carrier protein, mitochondrial OS=Homo sapiens OX=9606 GN=SLC25A3 PE=1 SV=2 | 40.095 |
| Q00610 | CLTC | Clathrin heavy chain 1 OS=Homo sapiens OX=9606 GN=CLTC PE=1 SV=5 | 191.615 |
| Q00839 | HNRNPU | Heterogeneous nuclear ribonucleoprotein U OS=Homo sapiens OX=9606 GN=HNRNPU PE=1 SV=6 | 90.584 |
| Q02790 | FKBP4 | Peptidyl-prolyl cis-trans isomerase FKBP4 OS=Homo sapiens OX=9606 GN=FKBP4 PE=1 SV=3 | 51.805 |
| Q02878 | RPL6 | 60S ribosomal protein L6 OS=Homo sapiens OX=9606 GN=RPL6 PE=1 SV=3 | 32.728 |
| Q04446 | GBE1 | 1,4-alpha-glucan-branching enzyme OS=Homo sapiens OX=9606 GN=GBE1 PE=1 SV=3 | 80.474 |
| Q06830 | PRDX1 | Peroxiredoxin-1 OS=Homo sapiens OX=9606 GN=PRDX1 PE=1 SV=1 | 22.11 |
| Q07020 | RPL18 | 60S ribosomal protein L18 OS=Homo sapiens OX=9606 GN=RPL18 PE=1 SV=2 | 21.634 |
| Q07666 | KHDRBS1 | KH domain-containing, RNA-binding, signal transduction-associated protein 1 OS=Homo sapiens OX=9606 GN=KHDRBS1 PE=1 SV=1 | 48.227 |
| Q08211 | DHX9 | ATP-dependent RNA helicase A OS=Homo sapiens OX=9606 GN=DHX9 PE=1 SV=4 | 140.958 |
| Q08257 | CRYZ | Quinone oxidoreductase OS=Homo sapiens OX=9606 GN=CRYZ PE=1 SV=1 | 35.207 |
| Q08380 | LGALS3BP | Galectin-3-binding protein OS=Homo sapiens OX=9606 GN=LGALS3BP PE=1 SV=1 | 65.331 |
| Q12905 | ILF2 | Interleukin enhancer-binding factor 2 OS=Homo sapiens OX=9606 GN=ILF2 PE=1 SV=2 | 43.062 |
| Q13098 | GPS1 | COP9 signalosome complex subunit 1 OS=Homo sapiens OX=9606 GN=GPS1 PE=1 SV=4 | 55.537 |
| Q13813 | SPTAN1 | Spectrin alpha chain, non-erythrocytic 1 OS=Homo sapiens OX=9606 GN=SPTAN1 PE=1 SV=3 | 284.539 |
| Q13867 | BLMH | Bleomycin hydrolase OS=Homo sapiens OX=9606 GN=BLMH PE=1 SV=1 | 52.562 |
| Q14195 | DPYSL3 | Dihydropyrimidinase-related protein 3 OS=Homo sapiens OX=9606 GN=DPYSL3 PE=1 SV=1 | 61.963 |
| Q14344 | GNA13 | Guanine nucleotide-binding protein subunit alpha-13 OS=Homo sapiens OX=9606 GN=GNA13 PE=1 SV=2 | 44.05 |
| Q14697 | GANAB | Neutral alpha-glucosidase AB OS=Homo sapiens OX=9606 GN=GANAB PE=1 SV=3 | 106.874 |
| Q14974 | KPNB1 | Importin subunit beta-1 OS=Homo sapiens OX=9606 GN=KPNB1 PE=1 SV=2 | 97.17 |
| Q15084 | PDIA6 | Protein disulfide-isomerase A6 OS=Homo sapiens OX=9606 GN=PDIA6 PE=1 SV=1 | 48.121 |
| Q15293 | RCN1 | Reticulocalbin-1 OS=Homo sapiens OX=9606 GN=RCN1 PE=1 SV=1 | 38.89 |
| Q15365 | PCBP1 | Poly(rC)-binding protein 1 OS=Homo sapiens OX=9606 GN=PCBP1 PE=1 SV=2 | 37.498 |
| Q15392 | DHCR24 | Delta(24)-sterol reductase OS=Homo sapiens OX=9606 GN=DHCR24 PE=1 SV=2 | 60.101 |
| Q15758 | SLC1A5 | Neutral amino acid transporter B(0) OS=Homo sapiens OX=9606 GN=SLC1A5 PE=1 SV=2 | 56.598 |
| Q16222 | UAP1 | UDP-N-acetylhexosamine pyrophosphorylase OS=Homo sapiens OX=9606 GN=UAP1 PE=1 SV=3 | 58.769 |
| Q16563 | SYPL1 | Synaptophysin-like protein 1 OS=Homo sapiens OX=9606 GN=SYPL1 PE=1 SV=1 | 28.565 |
| Q16658 | FSCN1 | Fascin OS=Homo sapiens OX=9606 GN=FSCN1 PE=1 SV=3 | 54.53 |
| Q16881 | TXNRD1 | Thioredoxin reductase 1, cytoplasmic OS=Homo sapiens OX=9606 GN=TXNRD1 PE=1 SV=3 | 70.906 |
| Q5JRX3 | PITRM1 | Presequence protease, mitochondrial OS=Homo sapiens OX=9606 GN=PITRM1 PE=1 SV=3 | 117.413 |
| Q5T1M5 | FKBP15 | FK506-binding protein 15 OS=Homo sapiens OX=9606 GN=FKBP15 PE=1 SV=2 | 133.63 |
| Q6PIU2 | NCEH1 | Neutral cholesterol ester hydrolase 1 OS=Homo sapiens OX=9606 GN=NCEH1 PE=1 SV=3 | 45.808 |
| Q7KZF4 | SND1 | Staphylococcal nuclease domain-containing protein 1 OS=Homo sapiens OX=9606 GN=SND1 PE=1 SV=1 | 101.997 |
| Q86YZ3 | HRNR | Hornerin OS=Homo sapiens OX=9606 GN=HRNR PE=1 SV=2 | 282.39 |
| Q8N1G4 | LRRC47 | Leucine-rich repeat-containing protein 47 OS=Homo sapiens OX=9606 GN=LRRC47 PE=1 SV=1 | 63.473 |
| Q8NBJ5 | COLGALT1 | Procollagen galactosyltransferase 1 OS=Homo sapiens OX=9606 GN=COLGALT1 PE=1 SV=1 | 71.636 |
| Q8NBX0 | SCCPDH | Saccharopine dehydrogenase-like oxidoreductase OS=Homo sapiens OX=9606 GN=SCCPDH PE=1 SV=1 | 47.151 |
| Q92598 | HSPH1 | Heat shock protein 105 kDa OS=Homo sapiens OX=9606 GN=HSPH1 PE=1 SV=1 | 96.865 |
| Q92820 | GGH | Gamma-glutamyl hydrolase OS=Homo sapiens OX=9606 GN=GGH PE=1 SV=2 | 35.964 |
| Q92945 | KHSRP | Far upstream element-binding protein 2 OS=Homo sapiens OX=9606 GN=KHSRP PE=1 SV=4 | 73.115 |
| Q969V3 | NCLN | Nicalin OS=Homo sapiens OX=9606 GN=NCLN PE=1 SV=2 | 62.974 |
| Q96G03 | PGM2 | Phosphoglucomutase-2 OS=Homo sapiens OX=9606 GN=PGM2 PE=1 SV=4 | 68.283 |
| Q96G75 | RMND5B | E3 ubiquitin-protein transferase RMND5B OS=Homo sapiens OX=9606 GN=RMND5B PE=1 SV=1 | 44.414 |
| Q96IF1 | AJUBA | LIM domain-containing protein ajuba OS=Homo sapiens OX=9606 GN=AJUBA PE=1 SV=1 | 56.934 |
| Q96KN7 | RPGRIP1 | X-linked retinitis pigmentosa GTPase regulator-interacting protein 1 OS=Homo sapiens OX=9606 GN=RPGRIP1 PE=1 SV=2 | 146.682 |
| Q96QK1 | VPS35 | Vacuolar protein sorting-associated protein 35 OS=Homo sapiens OX=9606 GN=VPS35 PE=1 SV=2 | 91.707 |
| Q96TA1 | FAM129B | Niban-like protein 1 OS=Homo sapiens OX=9606 GN=FAM129B PE=1 SV=3 | 84.138 |
| Q99798 | ACO2 | Aconitate hydratase, mitochondrial OS=Homo sapiens OX=9606 GN=ACO2 PE=1 SV=2 | 85.425 |
| Q99832 | CCT7 | T-complex protein 1 subunit eta OS=Homo sapiens OX=9606 GN=CCT7 PE=1 SV=2 | 59.367 |
| Q9BXS5 | AP1M1 | AP-1 complex subunit mu-1 OS=Homo sapiens OX=9606 GN=AP1M1 PE=1 SV=3 | 48.587 |
| Q9H4A4 | RNPEP | Aminopeptidase B OS=Homo sapiens OX=9606 GN=RNPEP PE=1 SV=2 | 72.596 |
| Q9H9B1 | EHMT1 | Histone-lysine N-methyltransferase EHMT1 OS=Homo sapiens OX=9606 GN=EHMT1 PE=1 SV=4 | 141.466 |
| Q9HDC9 | APMAP | Adipocyte plasma membrane-associated protein OS=Homo sapiens OX=9606 GN=APMAP PE=1 SV=2 | 46.48 |
| Q9NQW7 | XPNPEP1 | Xaa-Pro aminopeptidase 1 OS=Homo sapiens OX=9606 GN=XPNPEP1 PE=1 SV=3 | 69.918 |
| Q9NYK5 | MRPL39 | 39S ribosomal protein L39, mitochondrial OS=Homo sapiens OX=9606 GN=MRPL39 PE=1 SV=3 | 38.712 |
| Q9NZM1 | MYOF | Myoferlin OS=Homo sapiens OX=9606 GN=MYOF PE=1 SV=1 | 234.709 |
| Q9P0Z9 | PIPOX | Peroxisomal sarcosine oxidase OS=Homo sapiens OX=9606 GN=PIPOX PE=1 SV=2 | 44.066 |
| Q9UBQ7 | GRHPR | Glyoxylate reductase/hydroxypyruvate reductase OS=Homo sapiens OX=9606 GN=GRHPR PE=1 SV=1 | 35.668 |
| Q9UJ70 | NAGK | N-acetyl-D-glucosamine kinase OS=Homo sapiens OX=9606 GN=NAGK PE=1 SV=4 | 37.376 |
| Q9ULV4 | CORO1C | Coronin-1C OS=Homo sapiens OX=9606 GN=CORO1C PE=1 SV=1 | 53.249 |
| Q9Y262 | EIF3L | Eukaryotic translation initiation factor 3 subunit L OS=Homo sapiens OX=9606 GN=EIF3L PE=1 SV=1 | 66.727 |
| Q9Y312 | AAR2 | Protein AAR2 homolog OS=Homo sapiens OX=9606 GN=AAR2 PE=1 SV=2 | 43.472 |
| Q9Y490 | TLN1 | Talin-1 OS=Homo sapiens OX=9606 GN=TLN1 PE=1 SV=3 | 269.767 |

**Additional file 1:** Table S3 Docking score of bufalin and receptor

| Receptor | Ligand | docking score（Kcal/mol） |
| --- | --- | --- |
| Annexin A2 | Bufalin | -5.55 |
| TUBb | Bufalin | -5.82 |
| DRP1 | Bufalin | -5.77 |
| HSPA9 | Bufalin | -6.30 |
| HSPA8 | Bufalin | -6.04 |
